# Supplementary material for: Aqueous alternating electrolysis prolongs electrode lifespans under harsh operation conditions
Source: Nat Commun. 2024 Jul 23;15:6208. doi: 10.1038/s41467-024-50519-2 (PMC11266351; doi:10.1038/s41467-024-50519-2)
Supplement: Supplementary file 1 — Supplementary Information [file 41467_2024_50519_MOESM1_ESM.pdf]

Supplementary Information

Aqueous alternating electrolysis prolongs electrode lifespans under harsh operation conditions

Jie Liang,[1,2] Jun Li,[2] Hongliang Dong,[3] Zixiaozi Li,[2] Xun He,[2] Yan Wang,[2] Yongchao Yao,[2] Yuchun Ren,[2] Shengjun Sun,[1] Yongsong Luo,[1] Dongdong Zheng,[1] Jiong Li,[4] Qian Liu,[5] Fengming Luo,[6] Tongwei Wu,[2*] Guang Chen,[7*] Xuping Sun,[1,2,6*] and Bo Tang[1,8*]

[1]College of Chemistry, Chemical Engineering and Materials Science, Shandong Normal University, Jinan 250014, Shandong, China. [2]Institute of Fundamental and Frontier Sciences, University of Electronic Science and Technology of China, Chengdu 610054, Sichuan, China. [3]Center for High Pressure Science and Technology Advanced Research, Shanghai 201203, China. [4]Shanghai Synchrotron Radiation Facility, Shanghai Advanced Research Institute, Chinese Academy of Sciences, Shanghai 201210, China. [5]Institute for Advanced Study, Chengdu University, Chengdu 610106, Sichuan, China. [6]Center for High Altitude Medicine, West China Hospital, Sichuan University, Chengdu 610041, Sichuan, China. [7]Shaanxi Key Laboratory of Chemical Additives for Industry, College of Chemistry and Chemical Engineering, Shaanxi University of Science & Technology, Xi'an 710021, Shaanxi, China. [8]Laoshan Laboratory, Qingdao 266237, Shandong, China.

[*]Correspondence and requests for materials should be addressed to T.W. (e-mail: twwu77@uestc.edu.cn) or G.C. (e-mail: chenandguang@163.com) or X.S. (e-mail: xpsun@uestc.edu.cn) or B.T. (e-mail: tangb@sdnu.edu.cn)

Supplementary Note 1 | More details/messages about this work and the experiments.

In order to give the reader a better understanding of our work, we provide additional explanatory notes on this work in the following aspects.

(1) It is beneficial to explore and investigate different possibilities for pulsed electrochemistry in itself since the parameters that can be changed in conventional DC electrolysis are extremely restricted. Pulsed electrochemistry is now beginning to be exploited increasingly in a variety of electrochemical applications such as artificial N_2 fixation (*Angew. Chem. Int. Ed.* **62**, e202217635 (2023)), CO_2 reduction (*Nat. Catal.* **5**, 259–267 (2022), *Joule* **5**, 1987–2026 (2021), and *J. Am. Chem. Soc.* 145, 26213–26221 (2023)), nitrate-to-ammonia reduction (*J. Am. Chem. Soc.* **145**, 6471–6479 (2023)), ammonia recovery from urine (*Water Res.* **251**, 121129 (2024)), urea electrosynthesis from CO_2 and nitrate (*Nat. Sustain.* DOI: 10.1038/s41893-024-01302-0 (2024)), uranium extraction from seawater (*Nat. Energy* **2**, 17007 (2017)), CO_2 capture (*Joule* **7**, 2107–2117 (2023)), electrosynthesis of primary arylamines from nitrite and arylboronic acids (*Nat. Commun.* **14**, 5088 (2023)), more organic synthesis processes like C–H phosphorylation (e.g., *Nat. Synt.* **2**, 172–181 (2023), *Angew. Chem. Int. Ed.* 62, e202309620 (2023)), and so on. As such, our work is not only an up-to-date reference in the field of water electrolysis, but undoubtedly another exploration of the pulsed electrochemistry systems for the production of value-added chemicals.

(2) The synergistic interaction between AMCs and Fe group element (i.e., Fe, Co, and Ni) ions that we have discovered under the pulsed electrolysis conditions may have informative and positive impacts in many different electrocatalytic fields, such as molecular catalyst-based N_2 reduction (*J. Am. Chem. Soc.* **145**, 19912–19924 (2023)), oxygen electrochemistry (*Chem. Mater.* **33**, 6299–6310 (2021)), conventional acidic water oxidation catalysis (*Angew. Chem. Int. Ed.* **60**, 18821 (2021), *Nat. Commun.* **13**, 3784 (2022)), supercapacitors (*Chin. Chem. Lett.* **25**, 269–272 (2014)), capacitive desalination (*Nanoscale* **12**, 7586–7594 (2020)), batteries (*Adv. Energy Mater.* **8**, 1800298 (2018), *Mater. Today* 68, 22–23 (2023)), electrodeposition techniques (*Acc. Chem. Res.* **56**, 1421–1432 (2023)), and low-temperature thermoelectric applications (*Nat. Mater.* **5**, 537–540 (2006)), and so forth.

(3) In fact, there are several strategies for achieving catalyst self-healing, and Pb is not required for the catalyst to operate in self-healing mode (*Angew. Chem. Int. Ed.* **60**, 15821 (2021)). However, this work by Simondson et al. (*Angew. Chem. Int. Ed.* **60**, 15821 (2021)) still used a Pb-containing catalyst, $[Co–Fe–Pb]O_x$, to operate in acidic electrolytes in the absence of dissolved Pb^{2+}. The work again proves the importance of Pb for improving electrode stability in acidic solution. In fact, many previous studies (for instance, please refer to *Chem. Sci.* **8**, 4779–4794 (2017) and *Proc. Natl Acad. Sci.* **117**, 16187–16192 (2020)) have demonstrated that Pb is an effective element to enhance the electrode stability in acidic solution, whether it is introduced into the catalyst at the first place (*Angew. Chem. Int. Ed.* **60**, 15821 (2021)) or added to the electrolyte later (Fig. 1a), which involves the use of Pb, a known toxic element. AE-based methods in our study not only establish a new reliable strategy to repair electrode but also paves the way for the development of AE-based systems for the production of value-added chemicals, AMC-based electrocatalytic systems, and AMC-based electrodeposition technique, and beyond.

(4) In general, the higher the current density to be achieved by conventional water electrolysis, the greater the localized acidity as well as more intense bubble shock for the anode produced. The current densities in the stability tests performed by many previous literature on the design of acidic OER catalysts are not large enough to achieve the 2000 mA cm^{-2} of our

work (e.g., 10 mA cm^{-2} for *Angew. Chem. Int. Ed.* **58**, 7631 (2019), 10 mA cm^{-2} for *Energy Environ. Sci.* **17**, 1885–1893 (2024), 10 mA cm^{-2} for *Angew. Chem. Int. Ed*. **62**, e202314185 (2023), 10 mA cm^{-2} for *Angew. Chem. Int. Ed.* **62**, e202308704 (2023), 10 mA cm^{-2} for *Nat. Commun.* **14**, 7644 (2023), 50 mA cm^{-2} for *Adv. Mater.* DOI: 10.1002/adma.202314049 (2024), 100 mA cm^{-2} for *Angew. Chem. Int. Ed.* **63**, e202316903 (2024), 100 mA cm^{-2} for *Adv. Energy Mater.* DOI: 10.1002/aenm.202304479 (2024), 10 mA cm^{-2} for *Adv. Mater.* DOI: 10.1002/adma.202312608 (2024)). Therefore, these catalysts may decay more rapidly under high reaction currents.

(5) According to the characterization results, the coating on electrode surface that is eventually formed is practically amorphous species. Moreover, XANES data suggest that the oxidation state of cobalt in the coatings after different electrolysis times are different. Therefore, at this stage it is hard to precisely replenish the atoms by our strategy. The repair processes in the present work are more likely to form random, amorphous structures. Although we are currently unable to do so, the maintenance of defective electrode is theoretically possible, as many adjustable parameters (please refer to: *Nat. Synt.* **2**, 172–181 (2023)) of the pulsed AE electrochemistry as well as various solution compositions may enable the repair of the electrode defects at the atomic level of precision.

(6) In this work, saturated calomel electrodes (SCE, CHI 150) as the consumables are stored in a 50-mL centrifuge tube containing saturated potassium chloride solution when not in use for a short period of time (e.g., in two or three days), while we would cover and seal the bottom of the electrode (i.e., the ceramic liquid junction) with a plastic cap when not in use for a longer period of time. Not only do we need to maintain the moisture content of the ceramic core, but we also need to prevent crystallization from clogging the ceramic core.

(7) For reference electrode calibration, we performed the cyclic voltammetry test with a potential range near the open-circuit potential. Both the counter electrode and the reference electrode are commercial Pt plate-based electrodes. All the electrolytes are H_2-saturated acidic solution, and H_2 used for the calibration measurement comes from a specialized hydrogen generator (Supplementary Fig. 58c, SHC-300, SHANDONG SAIKESAISI Hydrogen Energy Co., Ltd, the website: http://www.ql-spe.com/portal/Article/index.html?cid=27&id=116).

(8) Future investigations will focus on improving the hydrogen generation efficiency, increasing current densities, employing lower concentrations of metal ions (or other alternative species), and developing matching equipment for more cost-effective and prolonged electrolysis based on the characteristics/properties of the reactions. For instance, the consumption of electrons must be allocated rationally as the formation of the protective layer consumes some electrons. A protective layer under ideal conditions would consume a very small amount of electrons or be formed by other means. Moreover, the formation and dissolution of this protective layer is close to 100% reversible. Currently, our system is not 100% reversible, but it is feasible to recover the metal ions in it. Future efforts are needed to achieve better performance by changing the parameters of the system (e.g., pH, temperatures, and pulsed programs) or using other redox pairs/deposition-dissolution species, or the devices, and so on. The present work is just a preliminary exploration.

(9) In fact, based on the AE processes, lower concentrations of metal salt can also achieve extended electrolysis lifespan. In order to speed up and streamline the process of determining the optimal ion combinations, we choose to employ excess AMCs and excess Fe group elemental ions in our exploratory work. In addition, for some ion combination conditions, the geometric area of the electrode may change in that the inner Ni is still in the process of non-

stop dissolution, and the final J is actually greater than $-2\&2$ A cm^{-2}. That being said, there are times when the nickel surface increases in area due to the derivation of coating species. Additionally, cutting can introduce some error.

Supplementary Note 2 | A more detailed explanation for Supplementary Figure 2

Rational design of catalysts including $W_{0.2}Er_{0.1}Ru_{0.7}O_{2-\delta}$ (*Nat. Commun.* **11**, 5368 (2020)), $Bi_xEr_{2-x}Ru_2O_7$ (*Nat. Commun.* **13**, 4106 (2022)), $Sr_{1-x}Na_xRuO_3$ (*Nat Commun*. **10**, 2041 (2019)), $Li_{0.52}RuO_2$ (*Nat. Commun.* **13**, 3784 (2022)), $Ru_5W_1O_x$ (*Nat. Commun.* 13, 4871 (2022)), Ni-RuO_2 (*Nat. Mater.* **22**, 100–108 (2023)), etc., are effective strategies to mitigate the acid etching-induced catalyst depletion.

However, as long as metal-based catalysts are used for OER in an acidic solution, the metal ion release issues cannot be completely avoided (Supplementary Fig. 2a). Accordingly, the oxygen evolution performance decay is inevitable (Supplementary Fig. 2b), which limits the development of acidic OER technologies. Progress in stabilizing metal anodes in HECs is still in early infancy, which encourages the electrochemistry communities to explore unconventional water electrolysis technologies, like Supplementary Fig. 1d. Unlike the development/screening of electrode materials with improved catalytic activity and durability, this work demonstrates a facile and effective approach for more durable oxygen evolution electrolysis in acids by “repairing” the catalyst periodically, promptly and uniformly, which leads to meaningful improvements in catalyst lifespan (Supplementary Fig. 2c and 2d). Specifically, the cations are re-electrodeposited to form porous coatings on the catalyst surface under the hydrogen evolution condition for a period of time at the end of each OER period. The electrodeposited structures are consumed during the OER process and regenerated again and again to maximize the lifespan of the anode catalyst. Ideally, the leaching rate and the deposition rate can be controlled to keep the electrolysis going, as the metal cations can always be deposited back to the catalysts.

Compared with previous oxygen evolution electrolysis in the acidic environment, the main advantages of our HBDT-involved intermittent oxygen evolution process are given below:

(i) Lifespan of electrocatalysts under HECs (such as acidic OER) can be greatly enhanced. Porous films of different metals can be prepared rapidly in a strongly acidic environment, due to the fact that H_2 bubbles act as soft templates in the process (*Acc. Chem. Res.* **56**, 1421–1432, (2023)). When H_2 evolution occurs, cations are simultaneously deposited onto the catalyst surface so that the electrode can endure longer electrolysis in acids.

(ii) From the perspective of electrolyte chemistry, the range of metal salts used in water electrolytes (and even other redox species) can be regulated is enormous. Moreover, the adjustable parameters of the alternating electrolysis technology itself are also very diverse (*Nat. Synth.* **2**, 172–181 (2023)). Therefore, the optimization of the performance that can be achieved by this technology is very promising in the future.

(iii) The production of H_2 is not affected by periodic switch of the electrical current polarity. Once time between each periodic polarity switch is slow enough, the H_2 and O_2 would not be mixed. Besides, we also present the possible device to better separate H_2-O_2, please see Fig. 6.

Supplementary Note 3 | Primary reasons for the final degradation of performance as well as why permanent alternating electrolysis cannot be achieved at this stage.

(1) Reason 1: We set maximum voltage limits (10 & −10 V) so that the electrochemical station program will automatically stop once the voltage required to achieve the current densities of 2 & −2 A cm^{-2} are too high. The applied electrode potentials are gradually increasing during the test, and finally a potential of 10 V can no longer support the electrode to attain the current density of 2 A cm^{-2}. At this point, electrolysis is terminated.
(2) Reason 2: The fact that nickel foam is running at extremely high current densities in an acidic solution makes it impossible to avoid coming into contact with the acids. Even with the external/outer protective layer, the internal nickel foam is still more or less etched by the acidic solution. At the end of our tests, sometimes the internal nickel foam had dissolved but the external protective layer was still there. This isolates the wires inside the electrode holder from the electrode. This breaks the circuit and thus stops the electrolysis process.
(3) Reason 3: Generally, the longer the electrolysis time, the thicker the external/outer protective layer becomes, and the excessive thickness of the protective layer is one of the reasons for the decay of activity. This is because the proper coating thickness is still difficult to maintain at this stage.
(4) Reason 4: Due to the technological limitations, this deposition-dissolution process is not fully reversible, which is similar to the charging and discharging process of a battery.

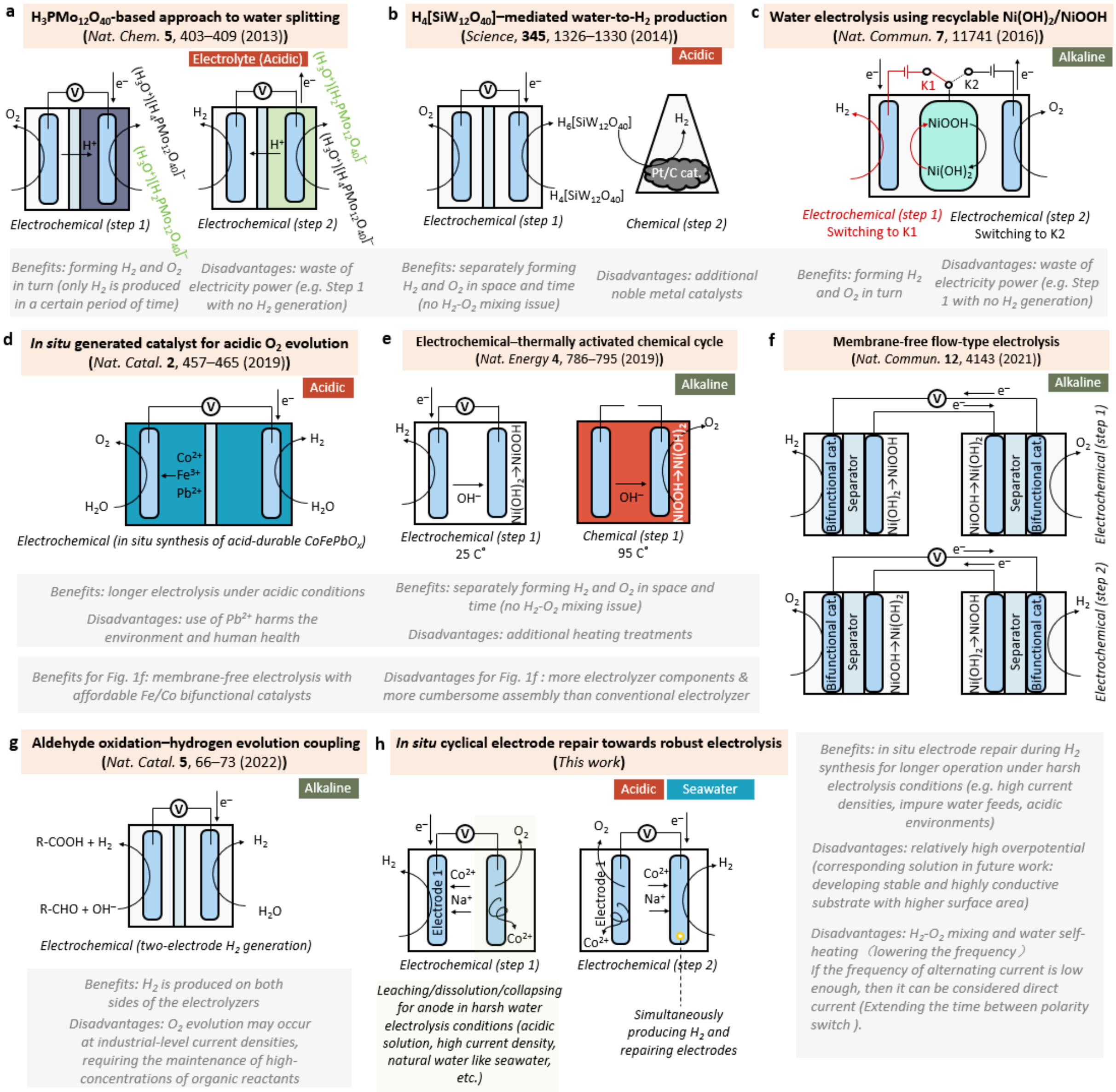

Supplementary Fig. 1 | Schemes of some representative unconventional water electrolysis technologies for H_2 production over the last decade (2013 to 2023). (a) H_2/O_2 evolution decoupling in water splitting using an electron-coupled-proton buffer. (b) Release of electrochemically produced H_2 from H_2 equivalents. (c) Using a redox medium to act as anode or cathode. (d) Adding Co^{2+}, Fe^{3+} and Pb^{2+} into the electrolyte to achieve a long-time acidic water electrolysis. (e) Electrochemical–thermally activated chemical water-splitting demonstration. (f) Membrane-free flow electrolyzer consisting of two sandwich-like compartments. (g) Adding furfural/5-hydroxymethylfurfural to the electrolyte as a reactant to accomplish an electricity-driven two-side H_2 production cell. (h) Electrolysis scheme for artificially repairing electrodes to achieve prolonged operation under harsh electrolytic conditions (e.g., acidic environments, high current densities, natural water sources). More examples can be are listed in Supplementary Table 2.

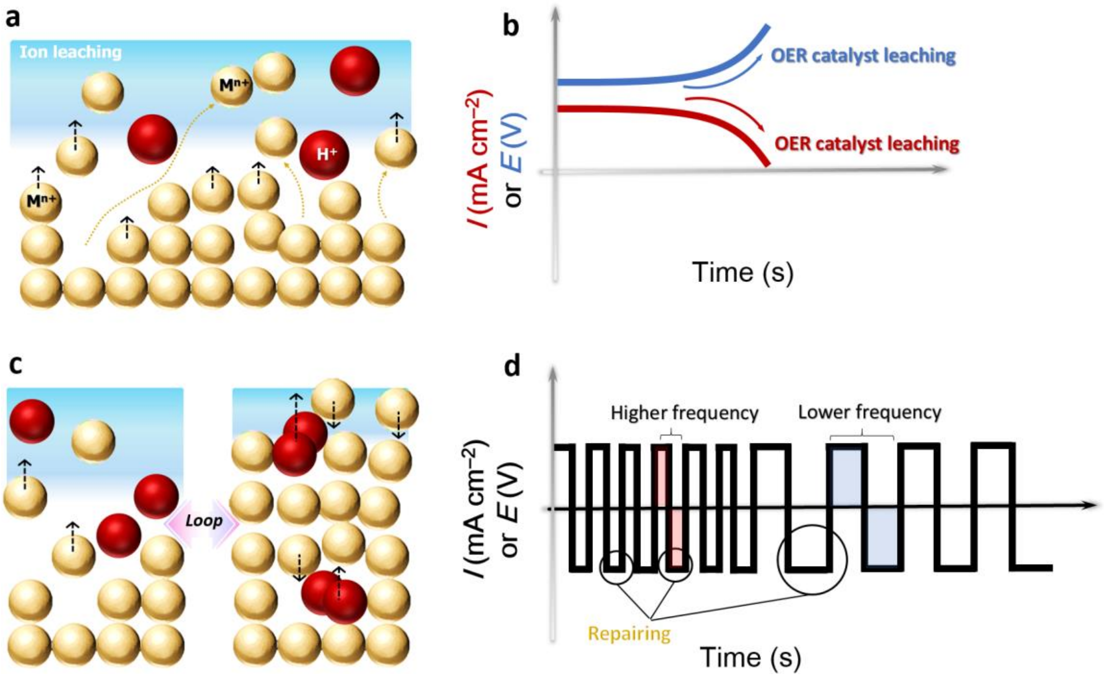

Supplementary Fig. 2 | Schematic diagrams to compare two different catalytic systems for oxygen evolution in the under HECs (such as electrolysis processes in the acidic media). (a,b) Conventional oxygen evolution electrolysis. (c,b) Dynamic hydrogen bubble template method (HBDT)-involved intermittent oxygen evolution.

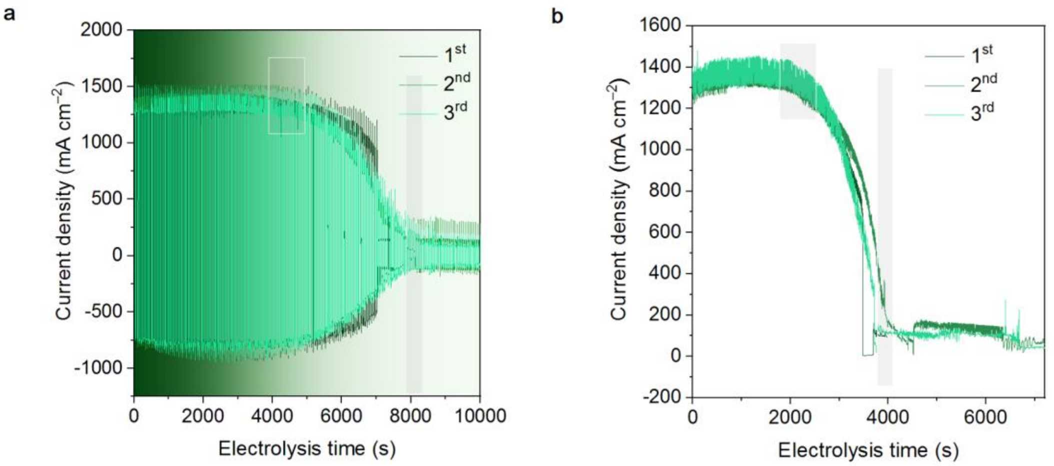

Supplementary Fig. 3 |. Durability tests for NiFE (a) with "repairing" steps (30s) and (b) without "repairing" steps. The two different electrolysis conditions were performed three times (*i.e.*, 1st, 2nd, 3rd in the figures). The geometric area of the exposed Ni foam electrode was between 0.24 cm^2 and 0.25 cm^2, and H_2SO_4 solution (pH < 1) was used as the electrolyte.

The actual electrolysis time of the NiFE with the "repairing" process (Supplementary Fig. 3a) is approximately twice as long as that of the Ni foam electrode without the "repairing" process (Supplementary Fig. 3b). The extra electrolysis time should be used for H_2 evolution, which means that the Ni foam electrode should suffer less corrosion under H_2 evolution conditions. At the same time, the actual O_2 evolution electrolysis time of NiFE is not significantly prolonged. This suggests that the electrolyte is too acidic, which leads to insignificant "repairing" effect. Therefore, it is necessary to reduce the acidity of the solution appropriately.

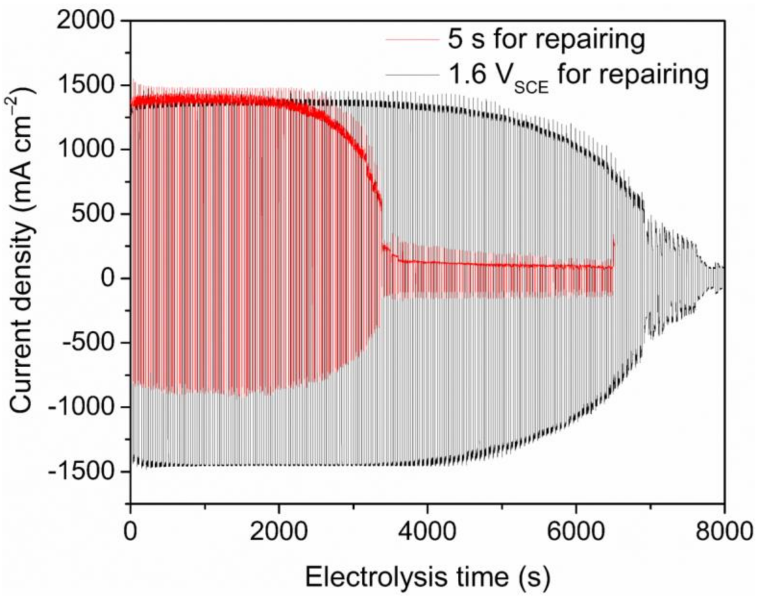

Supplementary Fig. 4 | Time-dependent electrolysis tests with a shorter step time of 5 s (red curves) and a higher applied potential of 1.6 V_{SCE} (black curves) for "repairing" catalyst and evolution of H_2.

As observed in **Supplementary Fig. 4**, after changing the step time from 30s to 5 s for "repairing" the catalyst, the catalyst lifespan shows no improvement (in comparison with **Supplementary Fig. 3b**). Moreover, increasing the applied potential at the time of "repairing" also show no significant enhancement in lifespan, again indicating that the dissolution rate of NiFE far exceeds the deposition rate.

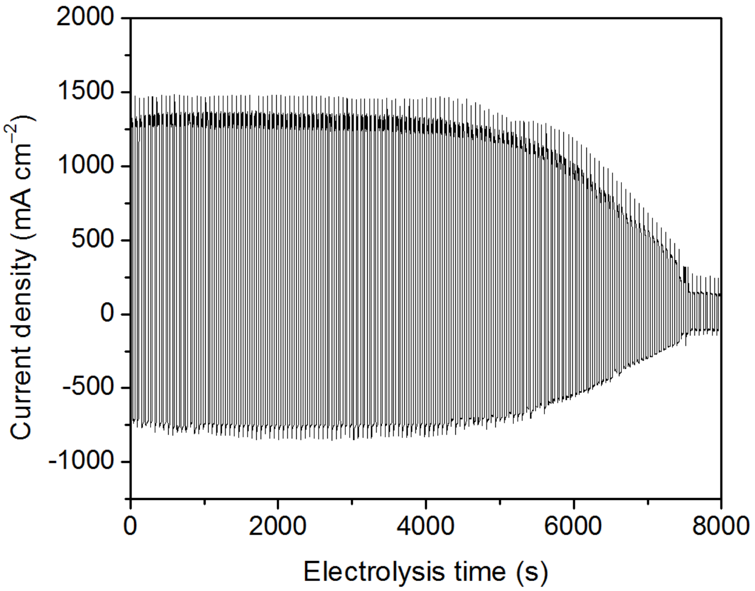

Supplementary Fig. 5 | Time-dependent electrolysis tests in 0.5 M H_2SO_4 solution with 0.1 M Ni^{2+} (step time for "repairing": 30 s). In comparison with data from Supplementary Fig. 3a, the catalyst lifespan improves little, implying the difficulty of "repairing" the Ni structures in highly acidic solution (pH<1). Slow deposition again emphasizes the importance of reducing solution pH.

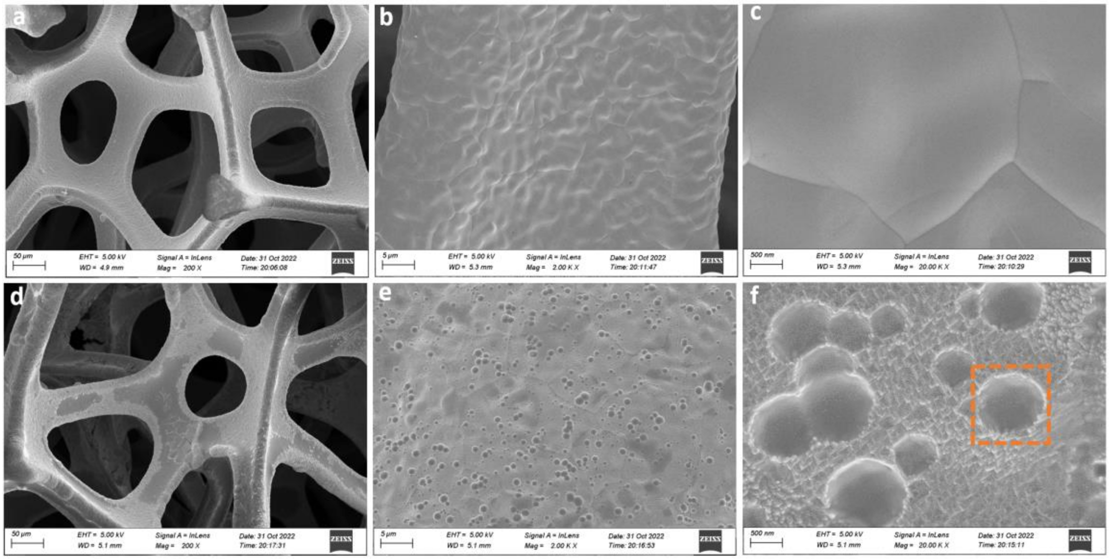

Supplementary Fig. 6 | SEM images of (a-c) pristine NiFE and (d-f) NiFE after cyclic time-dependent electrolysis tests in 0.5 M H_2SO_4 solution with 0.1 M Ni^{2+} for 700s (a step time of 30 s).

NiFE was badly etched after the cyclic electrolysis for 700s, and little deposited Ni species was left on the Ni substrate. The small and non-uniform pits on the Ni foam surface (Supplementary Fig. 6f) should be caused by the fast release of H_2 bubbles. The conclusion obtained from Supplementary Fig. 6 is consistent with that obtained from Supplementary Figs. 3–5.

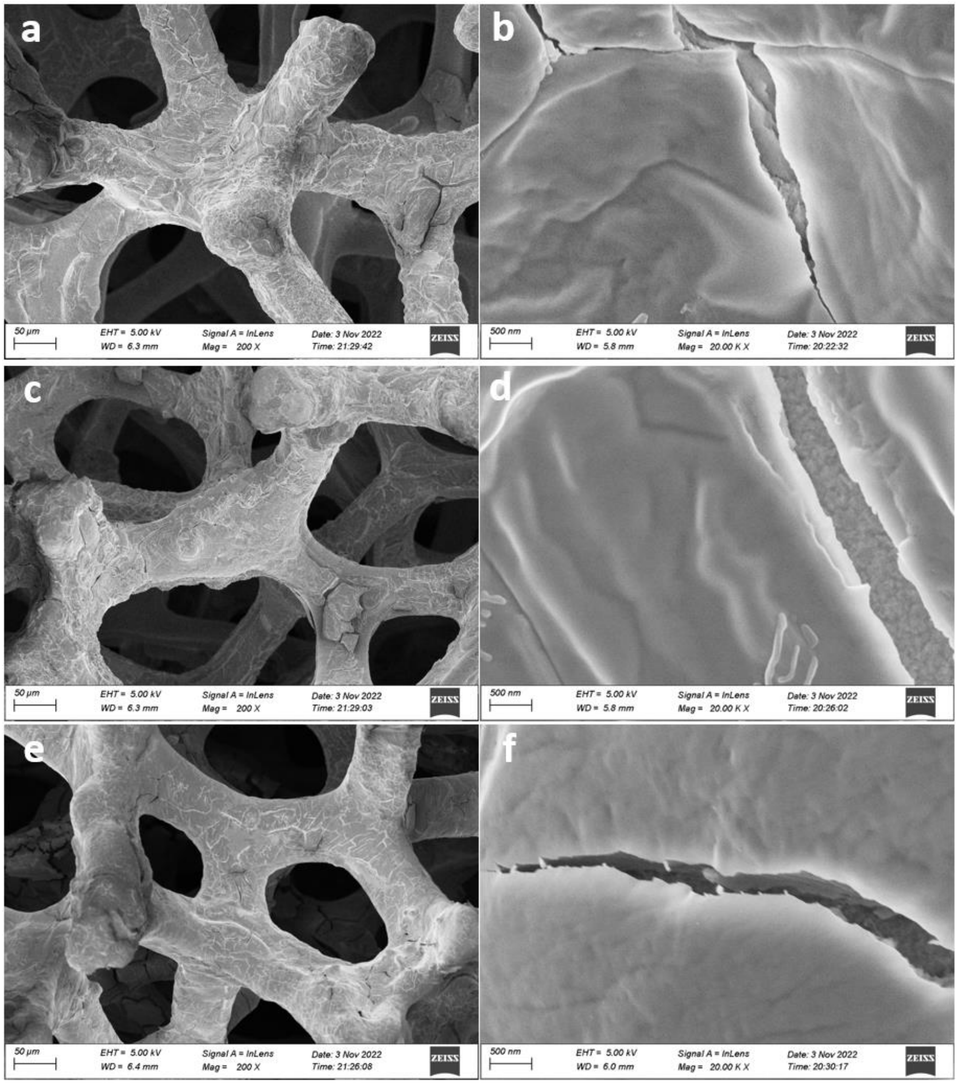

Supplementary Fig. 7 | SEM images of NiFE after cyclic time-dependent electrolysis tests for 720s (a step time of 30 s) in (a, b) pH >1 H_2SO_4 solution containing 0.1 M Ni^{2+}, (c, d) pH >1 H_2SO_4 solution containing 0.2 M Ni^{2+}, and (e, f) pH >1 H_2SO_4 solution containing 0.3 M Ni^{2+}.

As shown in Supplementary Fig. 7, Ni species are deposited on the NiFE after cyclic electrolysis for 720s, and cracks can be observed in the film. No notable pits caused by the fast release of H_2 bubbles can be observed on the NiFE. These images demonstrate that the repair step is successful after a slight increase in pH and that the catalyst possesses a stable shell after each repair process.

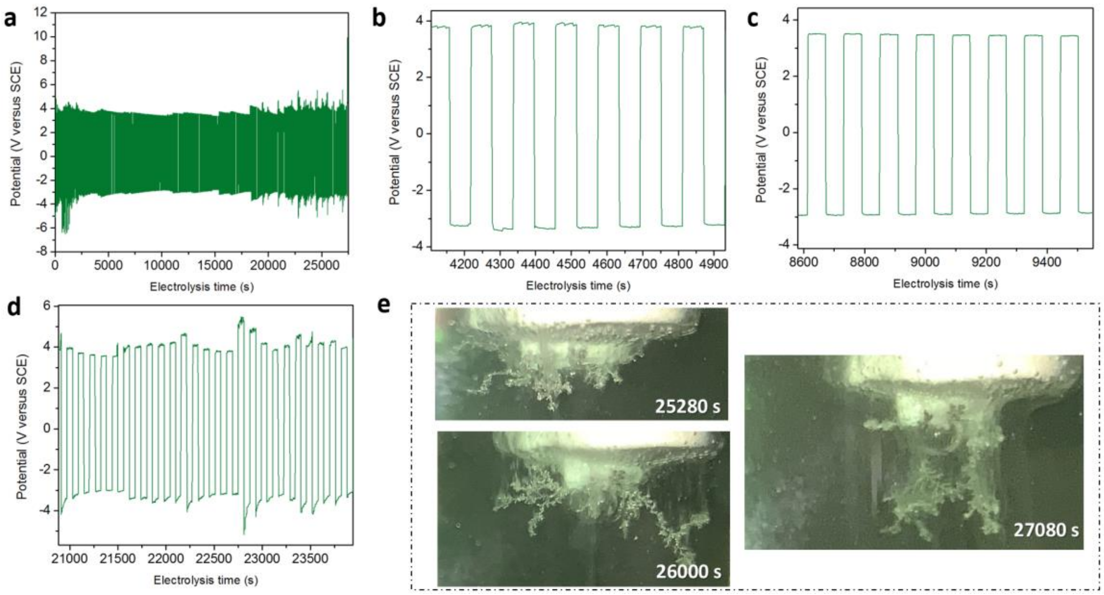

Supplementary Fig. 8 | "Time-dependent electrolysis curves in H_2SO_4 solution with 0.1 M Ni^{2+} and 0.1 M Fe^{3+} (1<pH<2), and corresponding optical photograph of the electrode near the end of electrolysis.

The fluctuation of the curve after 20000 s is because the NiFE is dissolving while still forming dendritic crystals, and the in situ generated bubbles are constantly hitting the electrode.

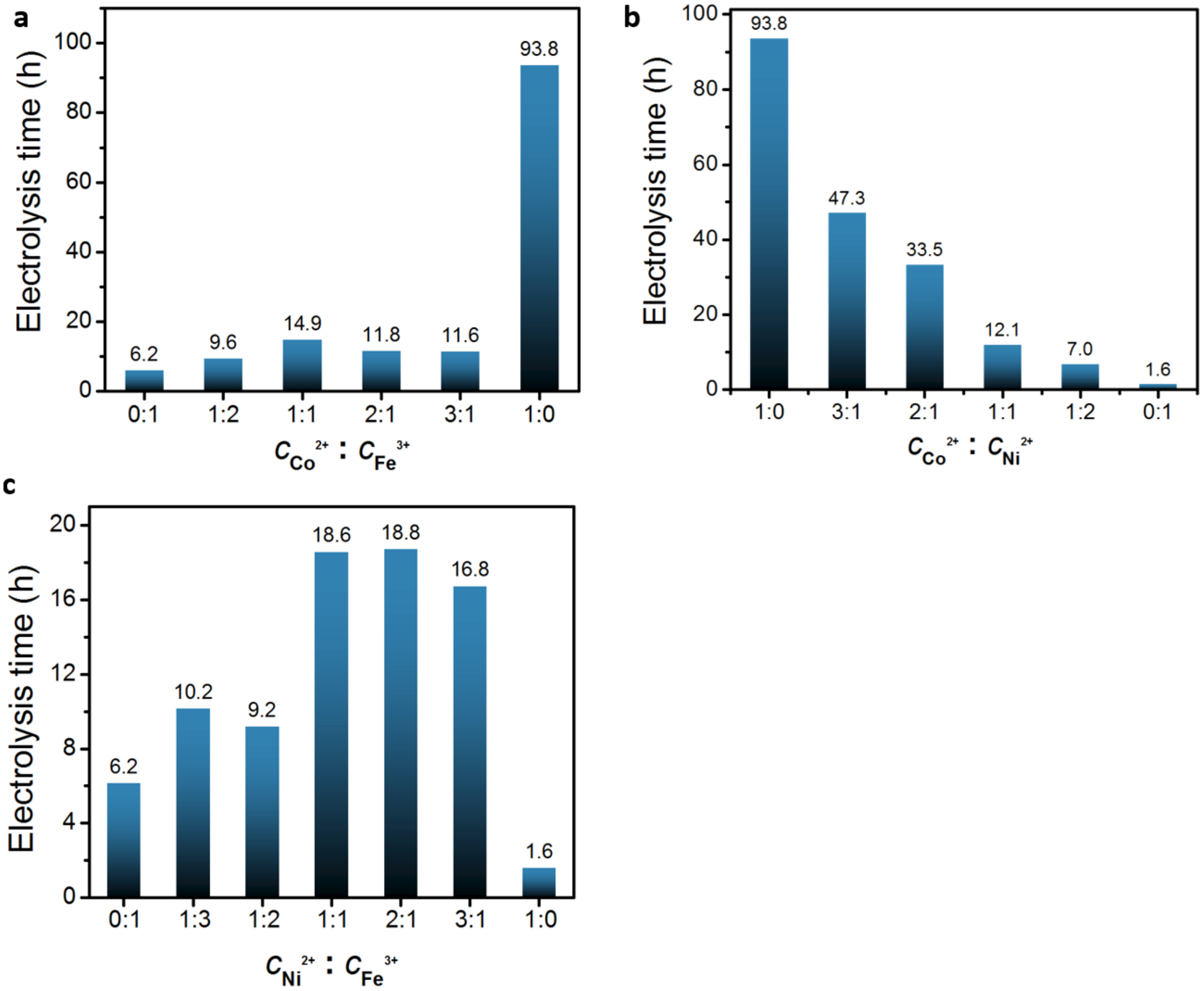

Supplementary Fig. 9 | Multimetallic systems with different content ratios of Fe-group ions for operation under fixed *J* of 2 A cm^{-2} and –2 A cm^{-2}. Concentration of M^{n+} is demoted as C_{Mn+}. Electrolytes for each test contains 1 M Na^{+}.

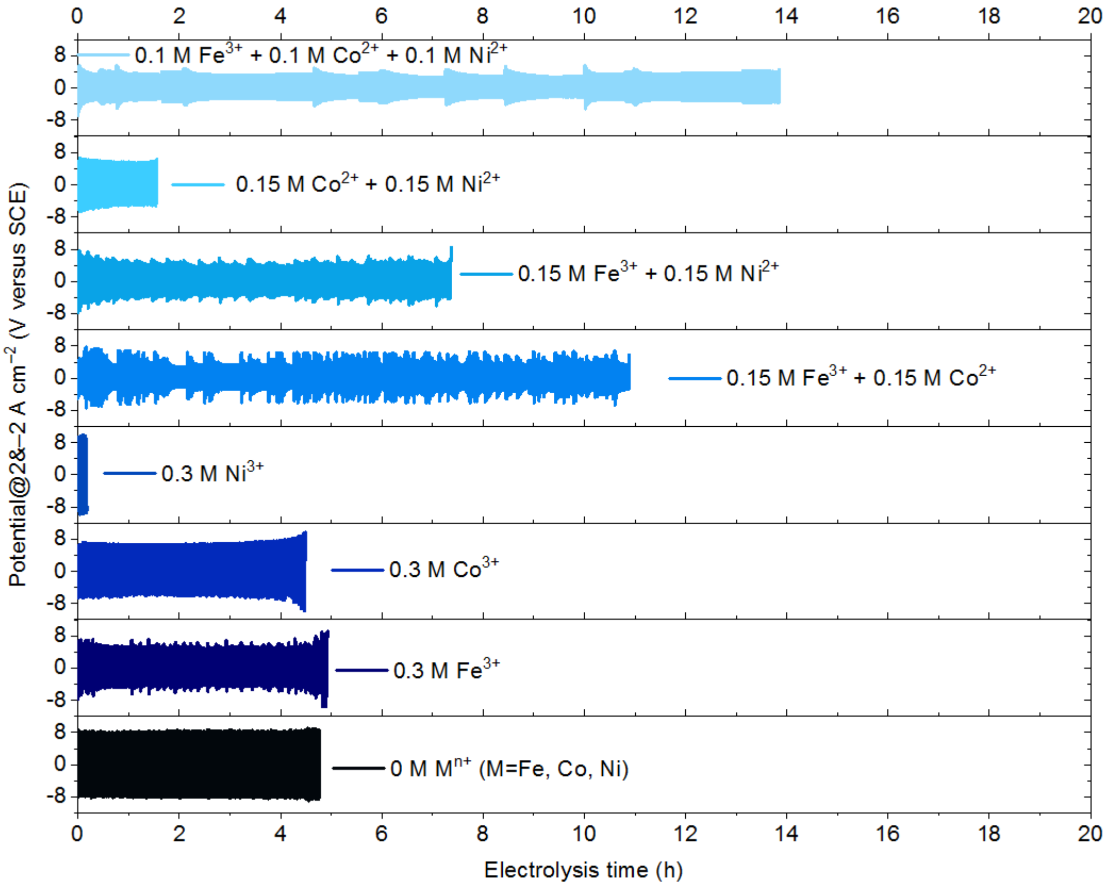

Supplementary Fig. 10 | Data of the first row of Fig. 3a in uncompressed form, showing alternating electrolysis results based on Fe group element ions.

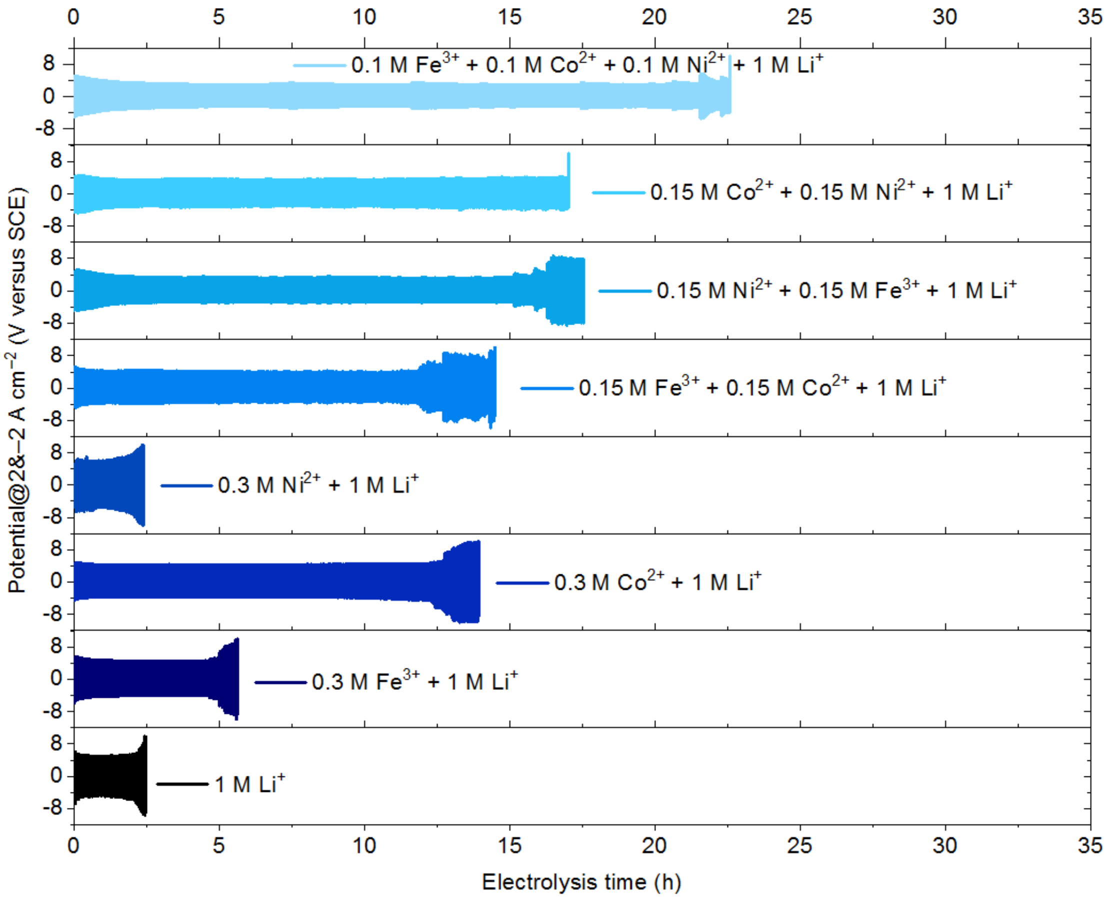

Supplementary Fig. 11 | Data of the second row of Fig. 3a in uncompressed form, showing alternating electrolysis results based on Li^+ alone and results based on Fe group element ions and Li^+.

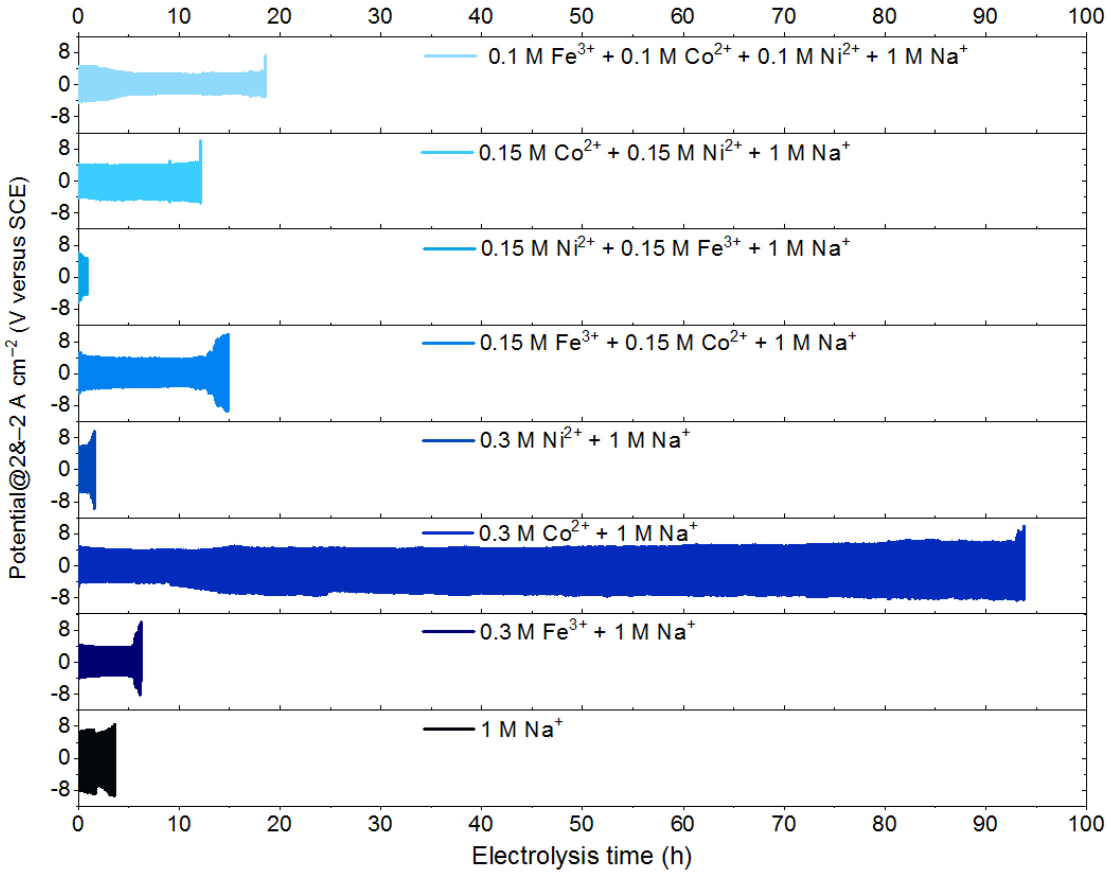

Supplementary Fig. 12 | Data of the third row of Fig. 3a in uncompressed form, showing alternating electrolysis results based on Na^+ alone and results based on Fe group element ions and Na^+.

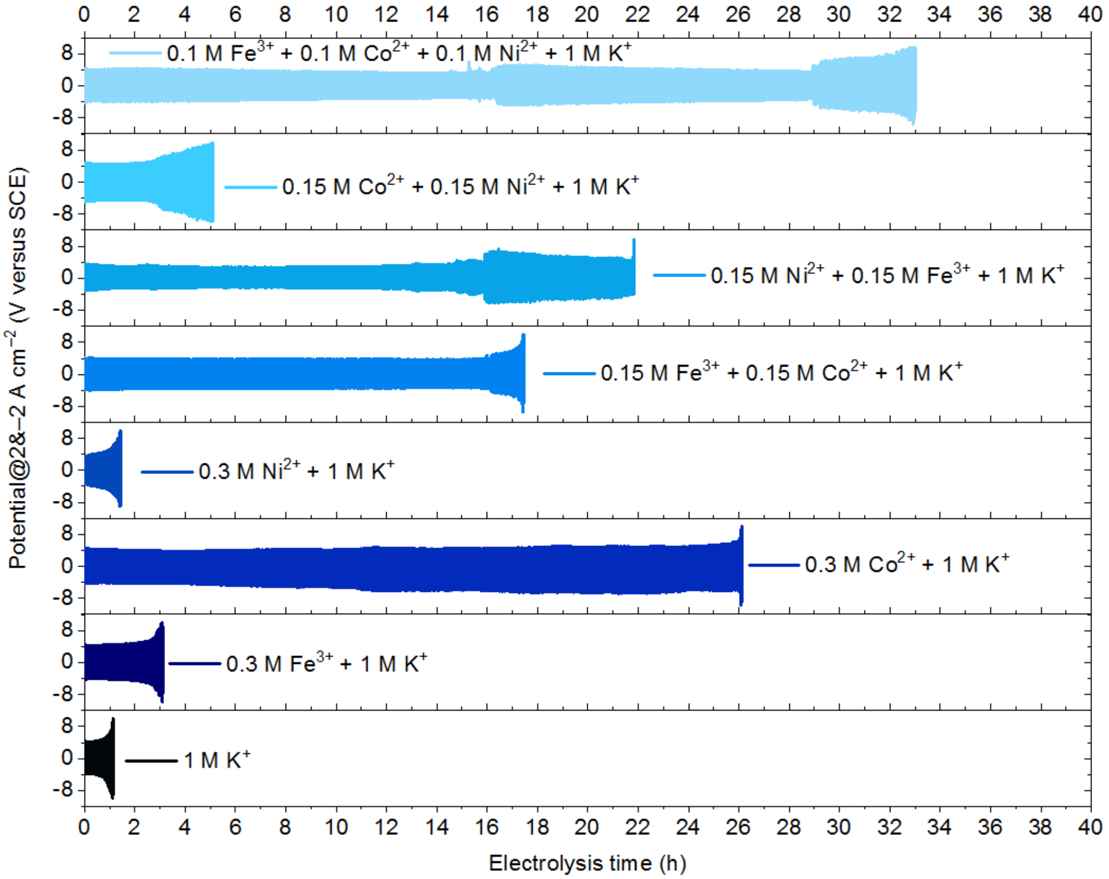

Supplementary Fig. 13 | Data of the fourth row of Fig. 3a in uncompressed form, showing alternating electrolysis results based on K^+ alone and results based on Fe group element ions and K^+.

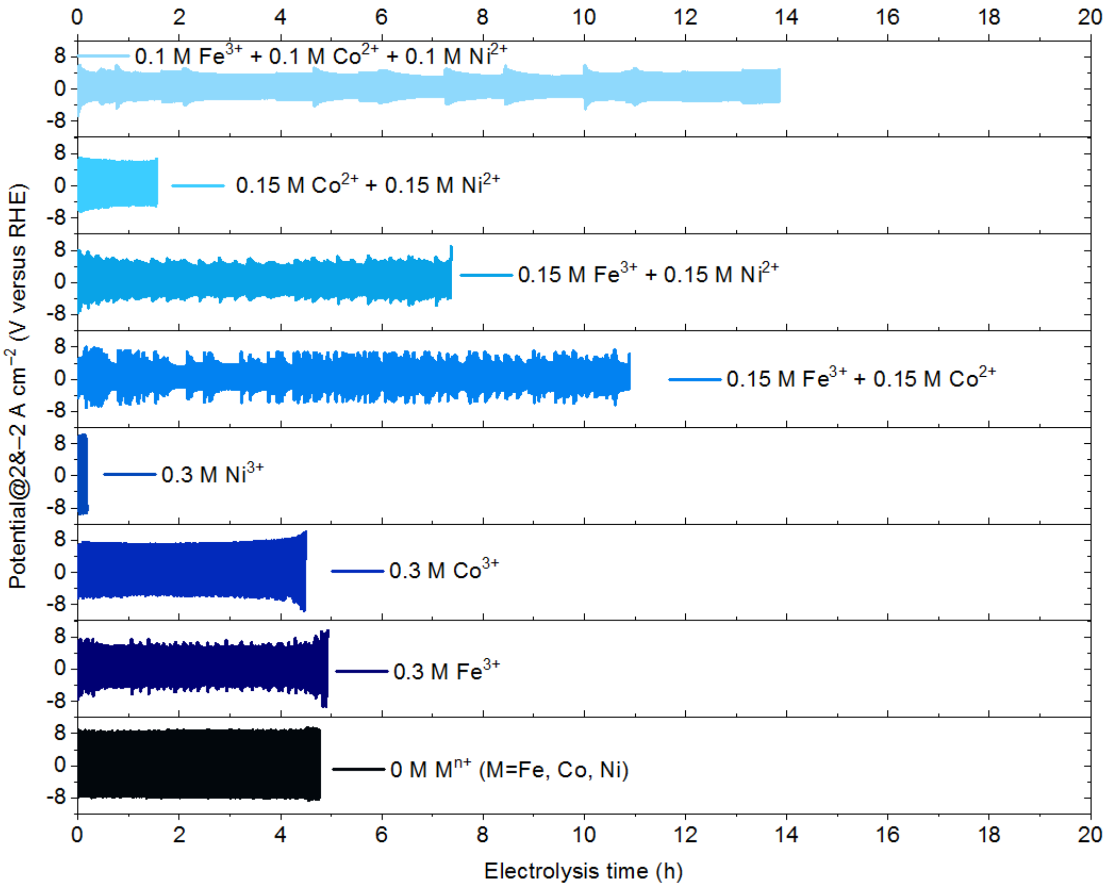

Supplementary Fig. 14 | Data of the first row of Fig. 3a in uncompressed form, showing alternating electrolysis results based on Fe group element ions. The potentials reported in this figure are relative to the RHE.

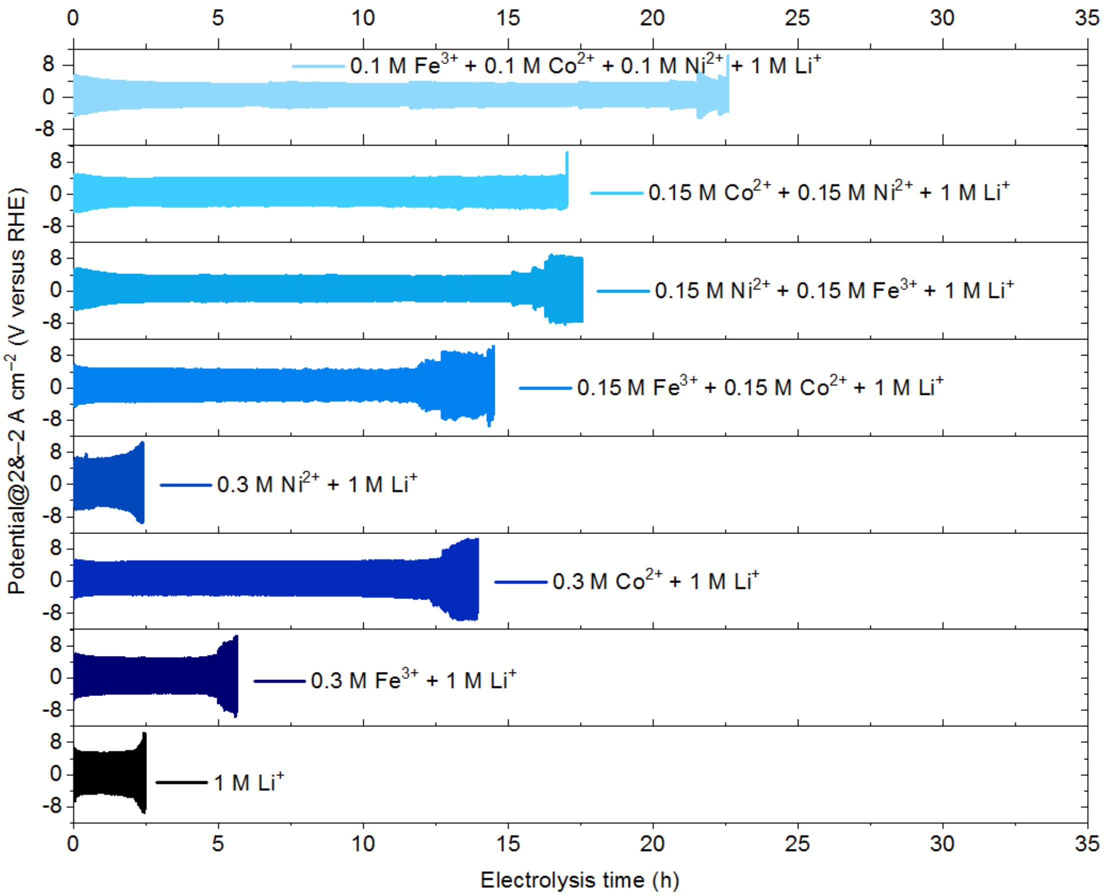

Supplementary Fig. 15 | Data of the second row of Fig. 3a in uncompressed form, showing alternating electrolysis results based on Li^+ alone and results based on Fe group element ions and Li^+. The potentials reported in this figure are relative to the RHE.

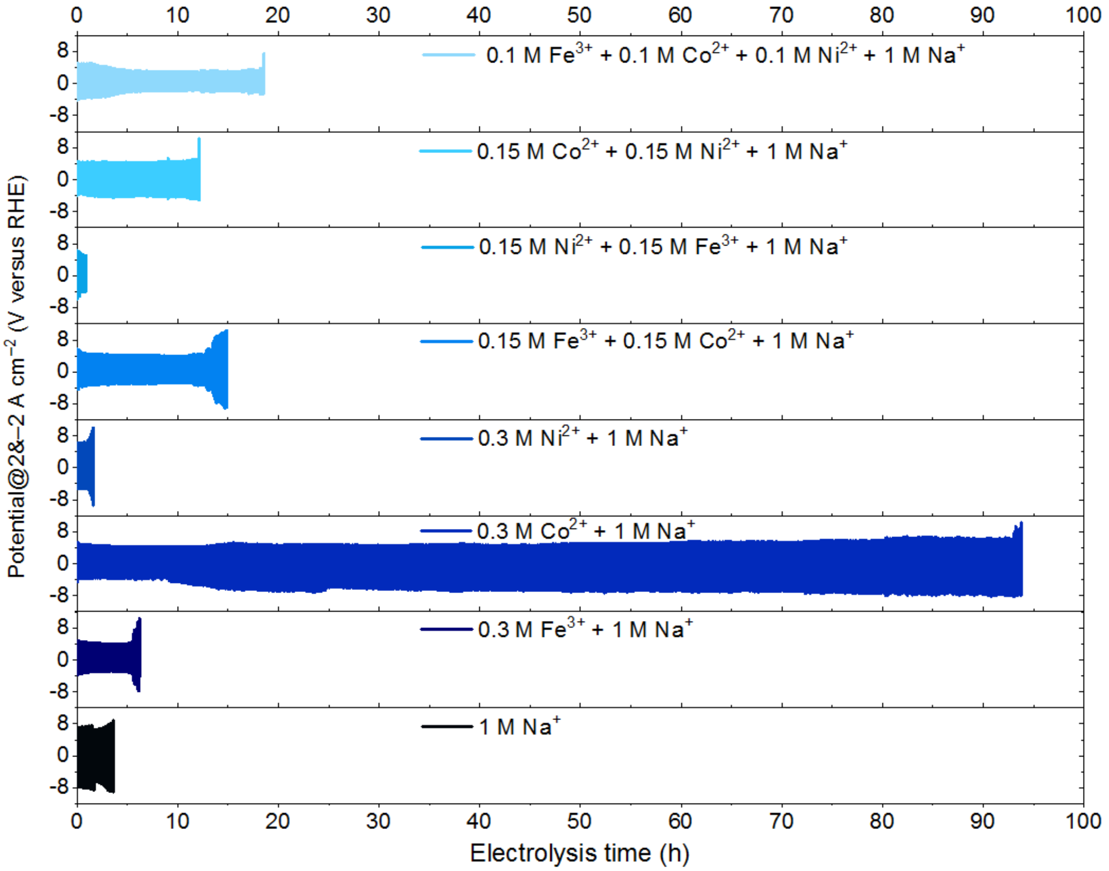

Supplementary Fig. 16 | Data of the third row of Fig. 3a in uncompressed form, showing alternating electrolysis results based on Na^+ alone and results based on Fe group element ions and Na^+. The potentials reported in this figure are relative to the RHE.

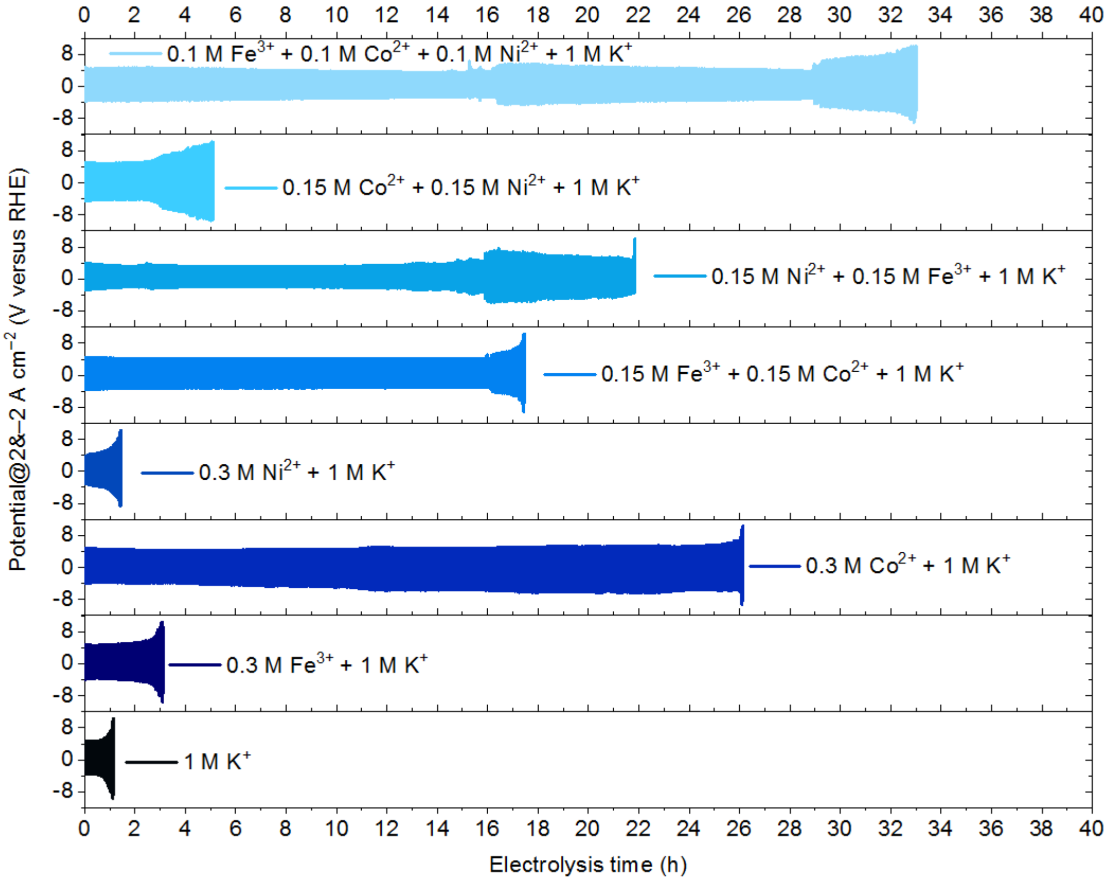

Supplementary Fig. 17 | Data of the fourth row of Fig. 3a in uncompressed form, showing alternating electrolysis results based on K^+ alone and results based on Fe group element ions and K^+. The potentials reported in this figure are relative to the RHE.

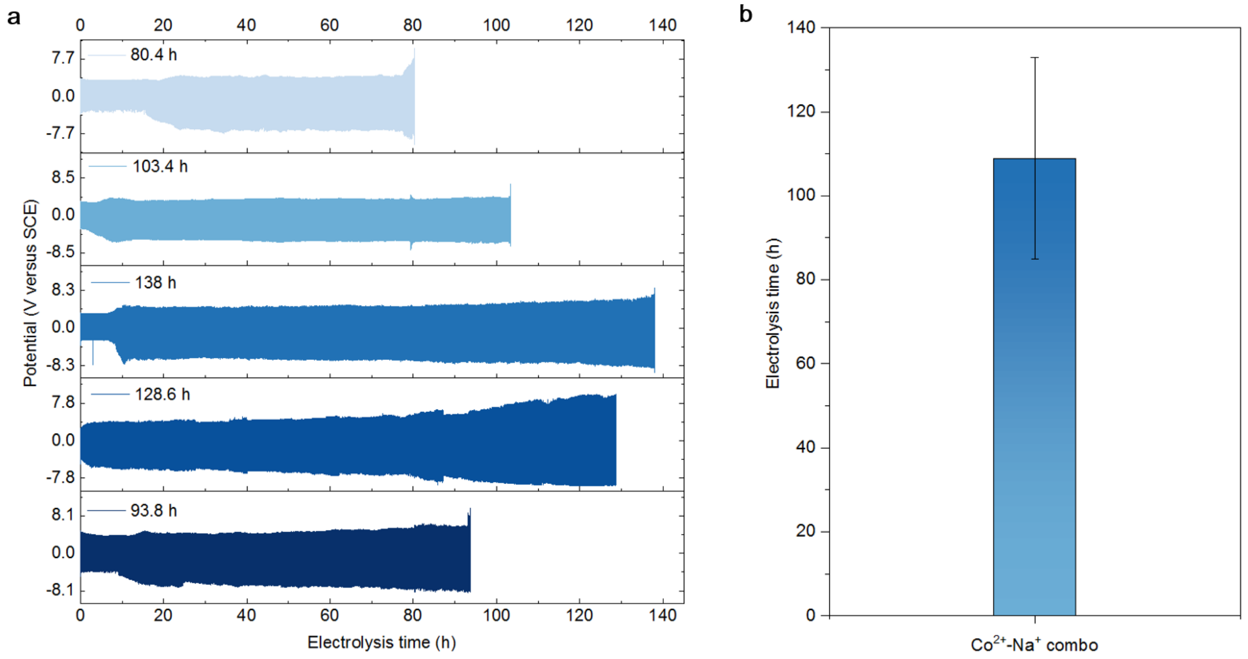

Supplementary Fig. 18 | Chronopotentiometric data in H_2SO_4 solution with Na^+ and Co^{2+} and the related electrolysis time. (a) Alternating electrolysis tests in an acidic solution based on the Na^+-Co^{2+} combo. (b) Average electrolysis time length. The average electrolysis lifespan is 108.84 ± 24 h. The error bar represents the results of different independent tests.

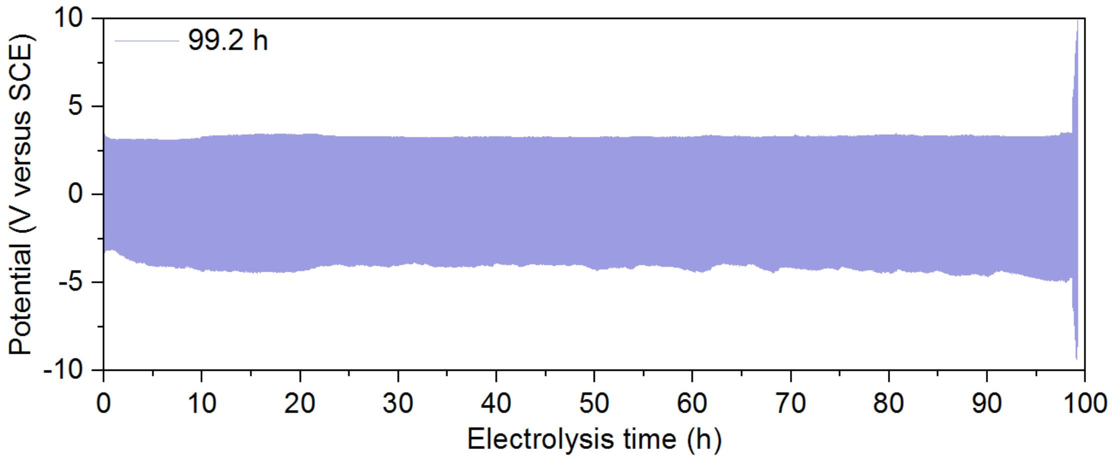

Supplementary Fig. 19 | Chronopotentiometric data based on the Co^{2+}-Na^{+} combo. Since the testing current densities were reduced to −1&1 A cm^{-2}, we reduced the corresponding Co^{2+} content to 0.2 M. Na^{+} levels remain in excess (1 M).

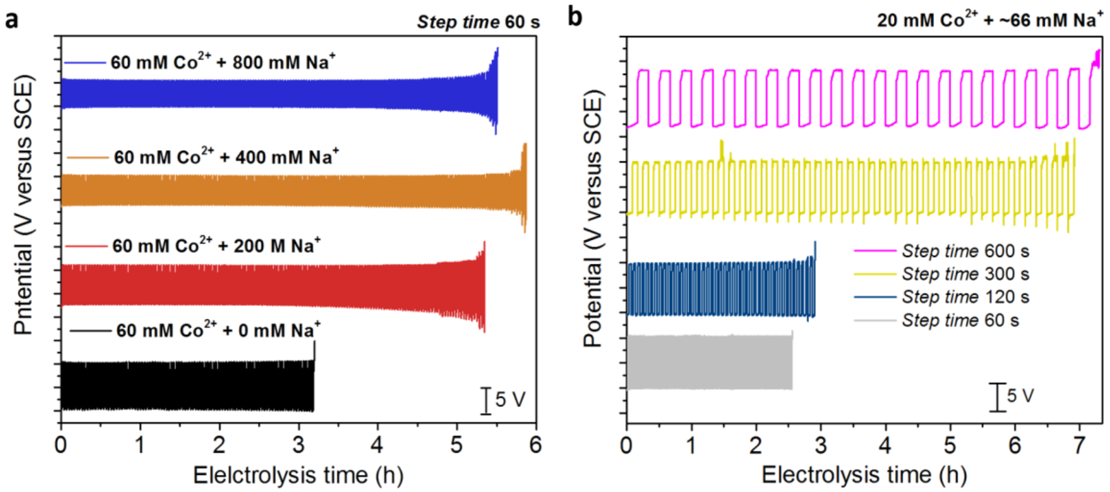

Supplementary Fig. 20 | Alternating electrolysis tests under the *J* of 1 A cm^{-2} and –1 A cm^{-2}. (a) The effect of Na^+-to-Co^{2+} relative ratio on the electrode lifetime. (b) The effect of step time (the frequency gradually extended from changing polarity every 1 minute to changing polarity every 10 minutes) on the electrode lifetime.

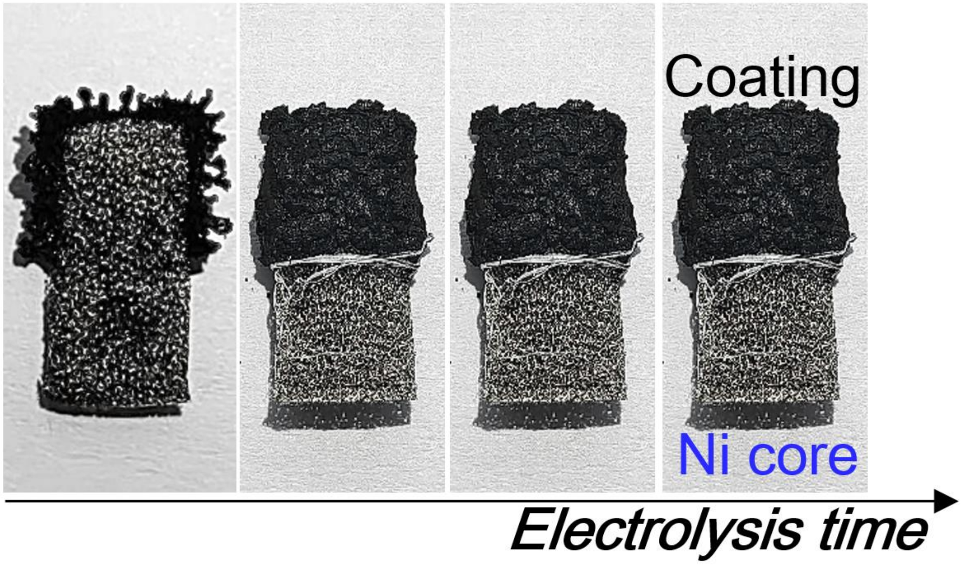

Supplementary Fig. 21 | Photos of the NiFE after prolonged a.c. electrolysis in acidic solution with Na^{+} and Co^{2+}.

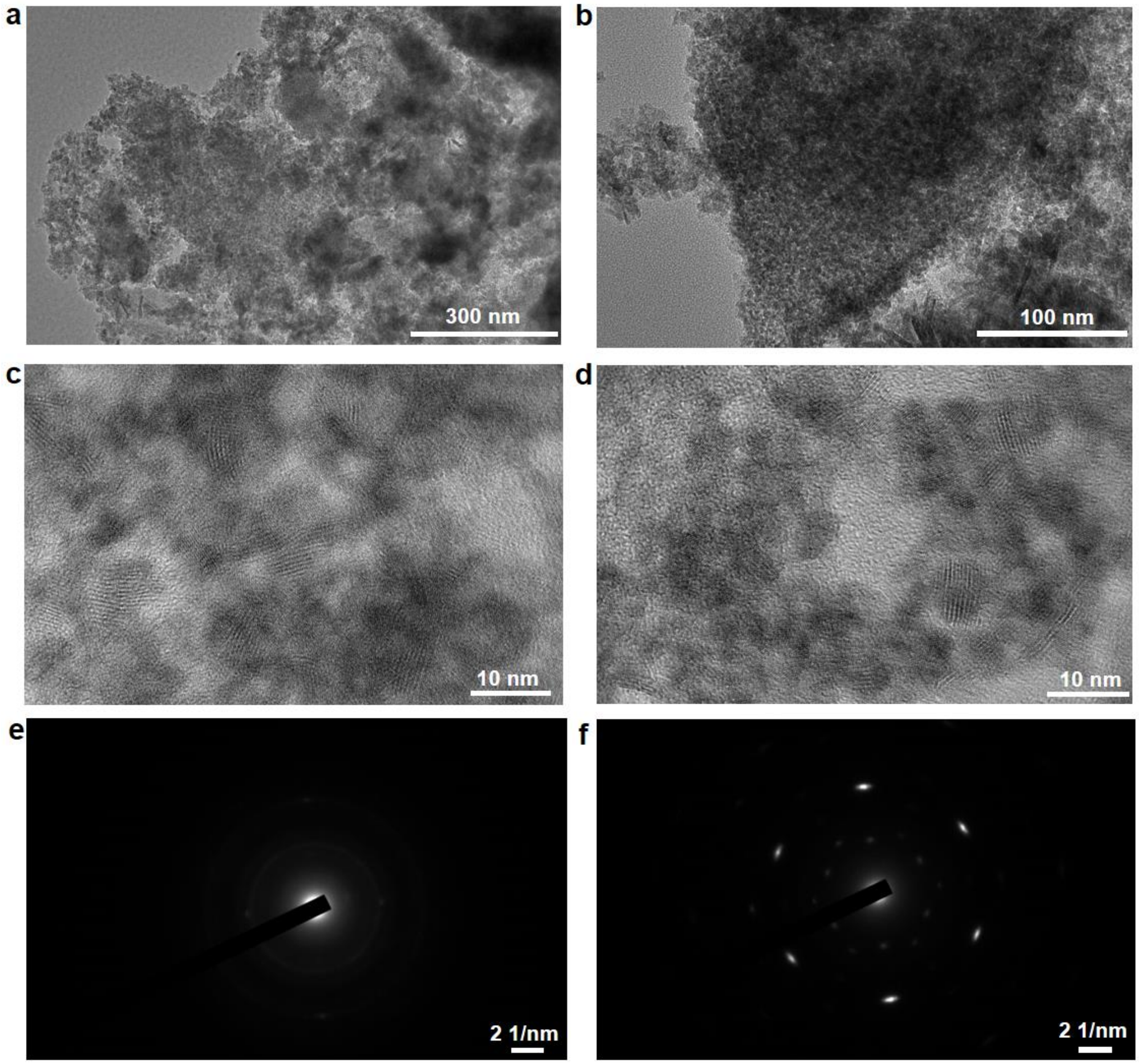

Supplementary Fig. 22 | (a,b) TEM, (c,d) HRTEM and (e,f) selected area electron diffraction (SAED) images of the in situ formed black coating after 30 h of alternating electrolysis (2 A cm^{-2} & −2 A cm^{-2}). The material consists of tiny crystalline nanoparticles and rich low-crystallinity/amorphous structures.

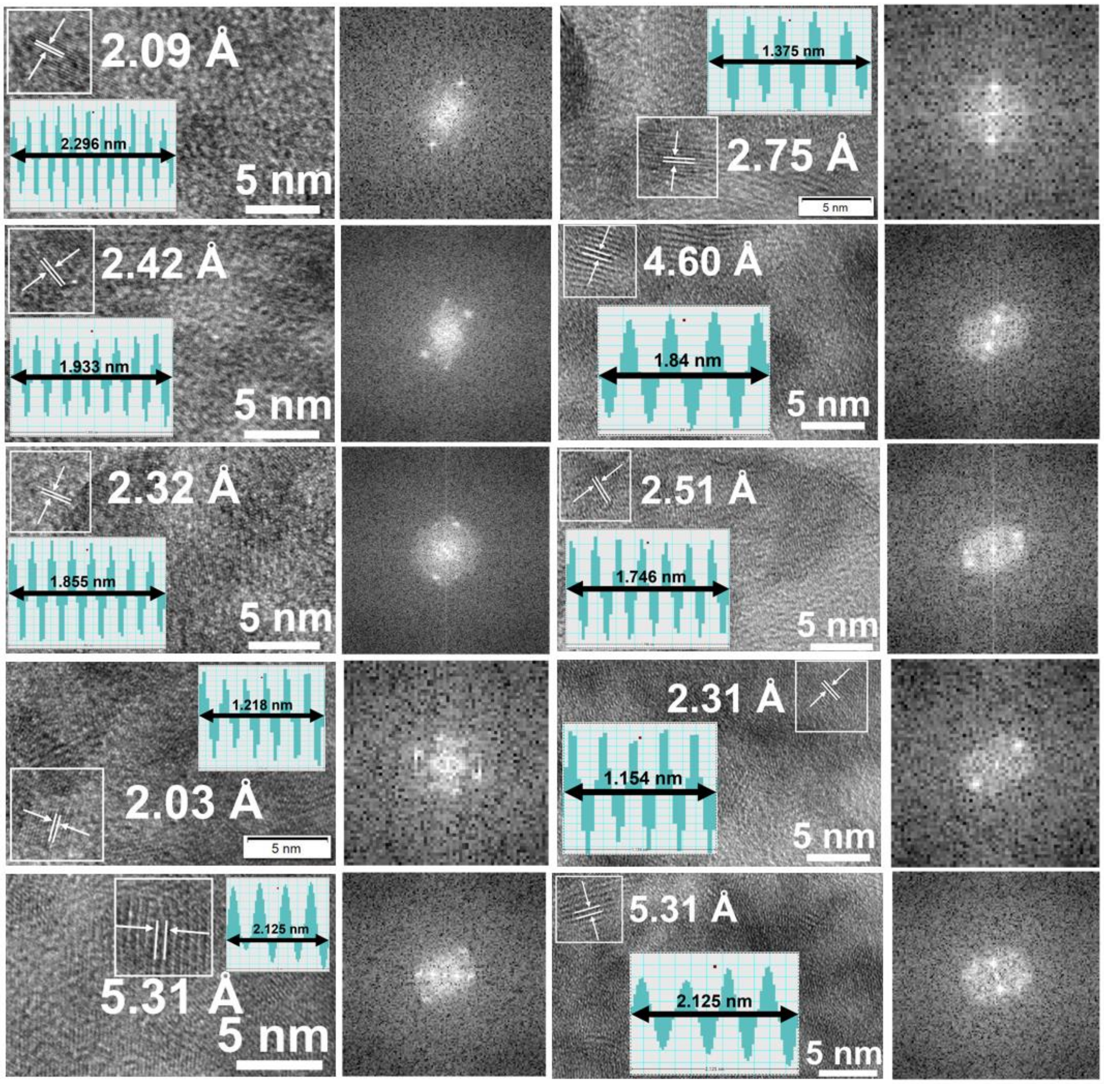

Supplementary Fig. 23 | HRTEM images of the in situ formed black coating after 30 h of alternating electrolysis, with fast Fourier transformation (FFT) images and the corresponding line scan of the square regions in HRTEM images.

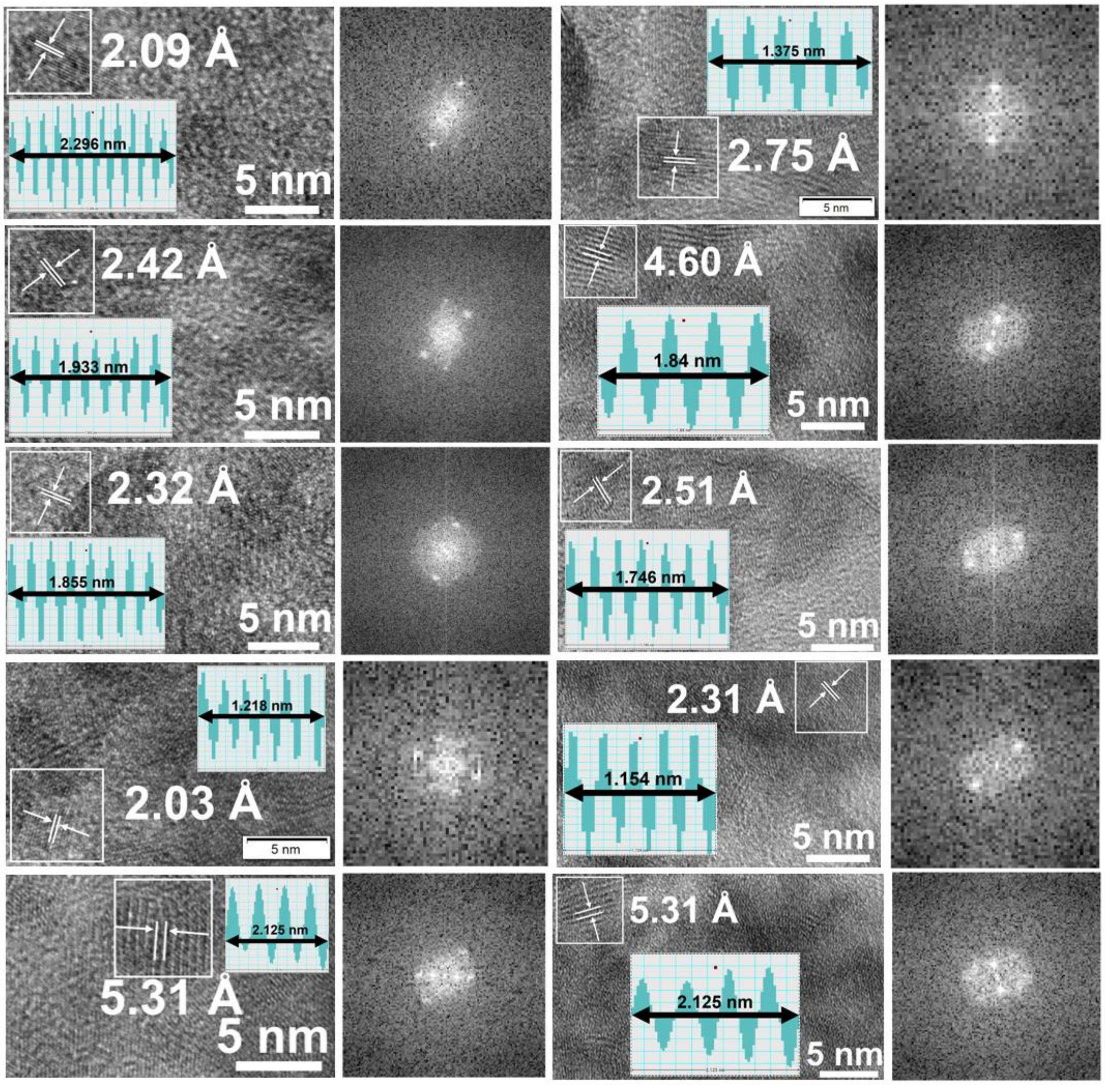

Supplementary Fig. 24 | More HRTEM images of the in situ formed black coating, with fast Fourier transformation (FFT) images and the corresponding line scan of the square regions in HRTEM images. According to results of Supplementary Figs. 22–24, it is clear that black coating consists of both crystalline and amorphous Co species. Based on lattice fringe spacing alone, possible crystalline Co species include metallic Co, Co_3O_4, $Co_2(OH)_3Cl$, CoOOH, etc., No lattice fringe for CoO can be found in HRTEM images of two independent samples.

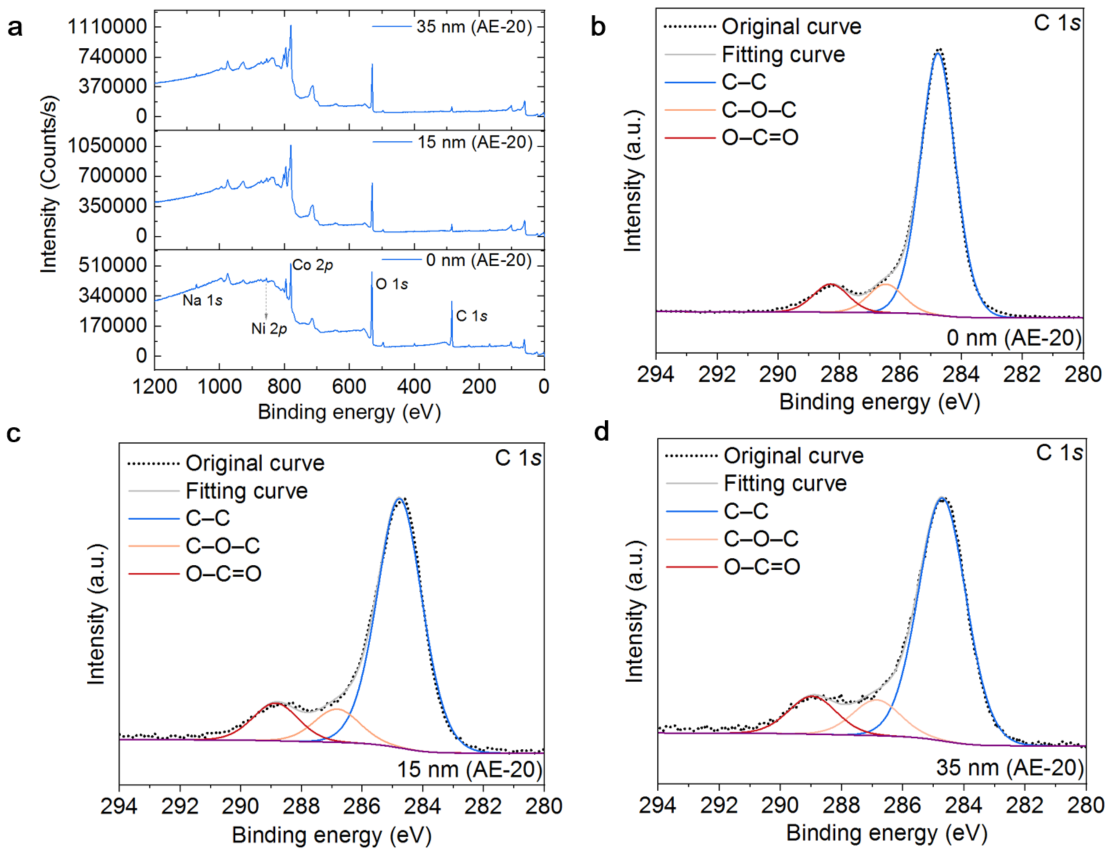

Supplementary Fig. 25 | (a) XPS survey spectra of the post-reaction electrodes after 20-h AE tests. (b-c) Depth-profiling XPS spectra in C 1*s* region, with both original data curves and related fitting data curves.

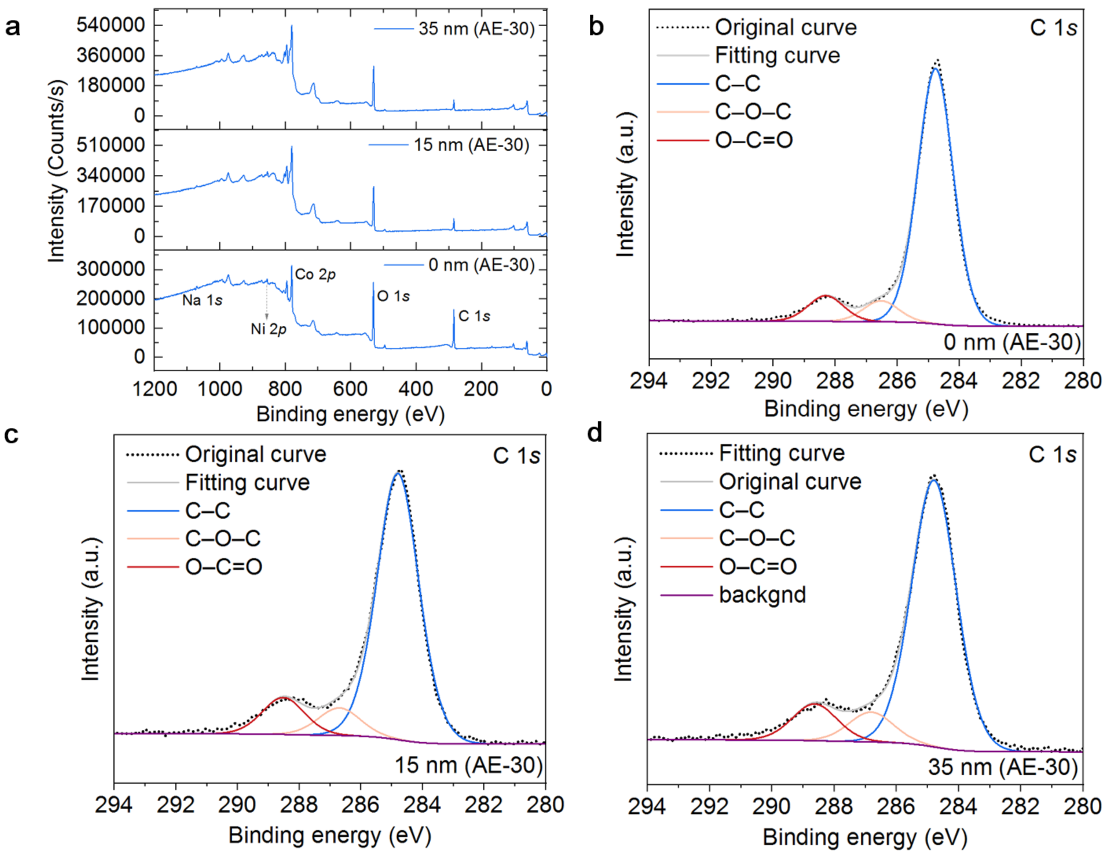

Supplementary Fig. 26 | (a) XPS survey spectra of the post-reaction electrodes after 30-h AE tests. (b-c) Depth-profiling XPS spectra in C 1*s* region, with both original data curves and related fitting data curves.

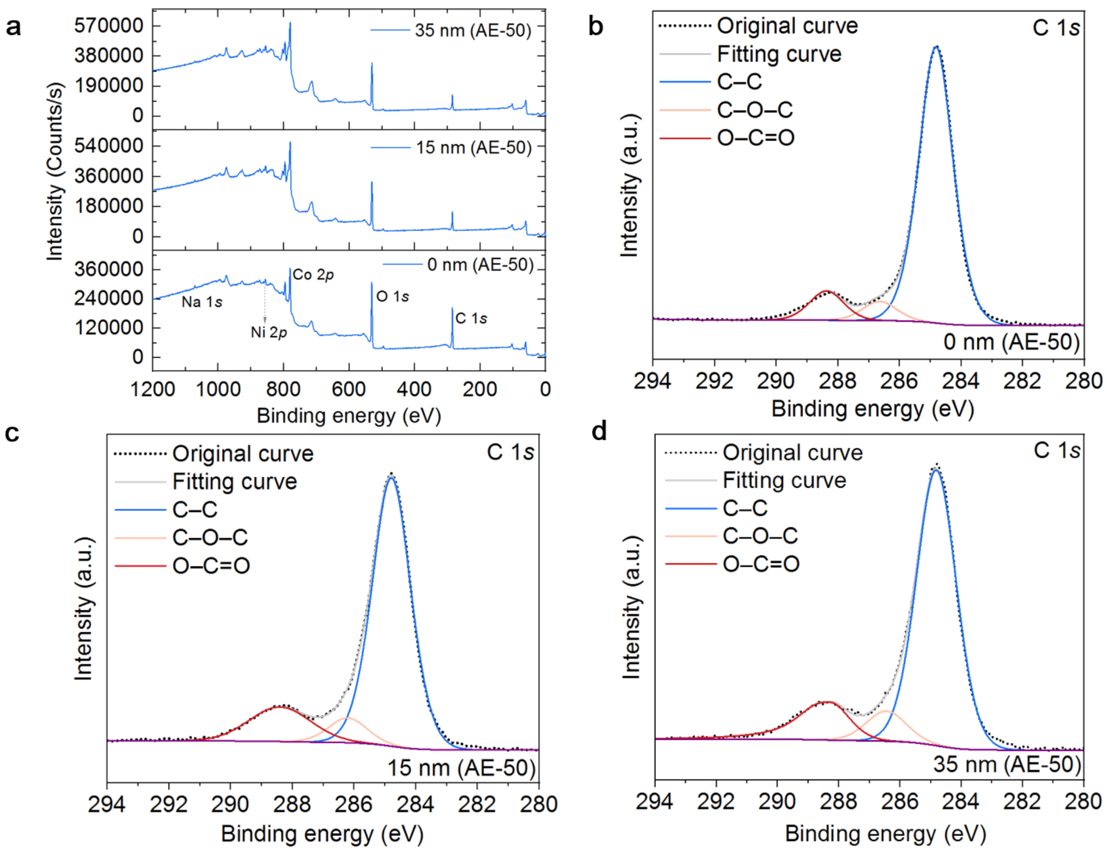

Supplementary Fig. 27 | (a) XPS survey spectra of the post-reaction electrodes after 50-h AE tests. (b-c) Depth-profiling XPS spectra in C 1*s* region, with both original data curves and related fitting data curves.

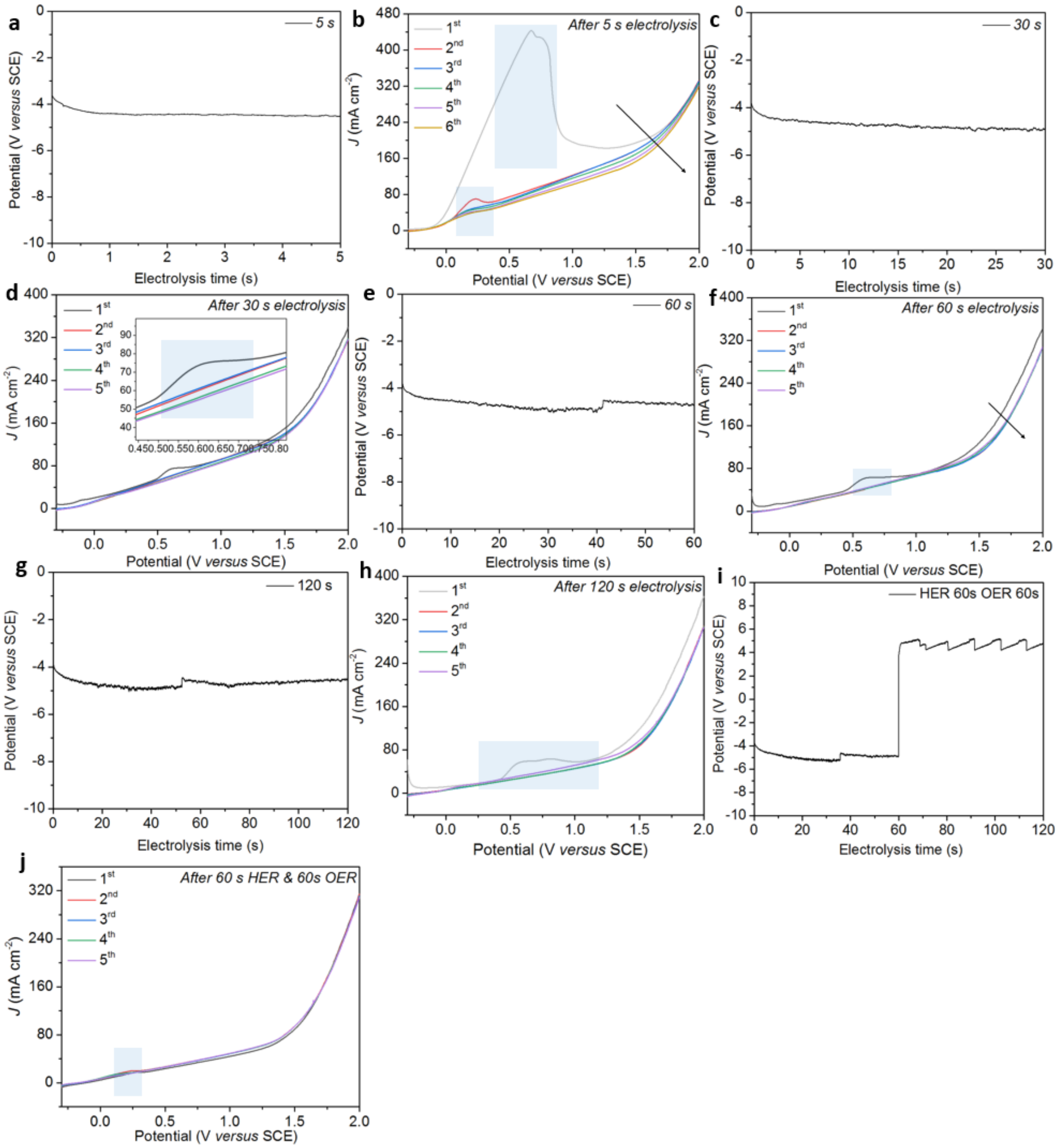

Supplementary Fig. 28 | Chronopotentiometric curves and the corresponding OER curves in 1.3 M Na^+. (a) *V*-t curve and (b) polarization curves after 5 s HER electrolysis. (c) *V*-t curve and (d) polarization curves after 30 s HER electrolysis. (e) *V*-t curve and (f) polarization curves after 60 s HER electrolysis. (g) *V*-t curve and (h) polarization curves after 120 s HER electrolysis. (i) *V*-t curve and (j) polarization curves after 60 s HER-60 s OER electrolysis. Supplementary Fig. 28b, 28d, 28f, 28h and 28j imply that the redox of the substrate (e.g. NiFE) itself may be going on and occurring all the time in the process of alternating electrolysis.

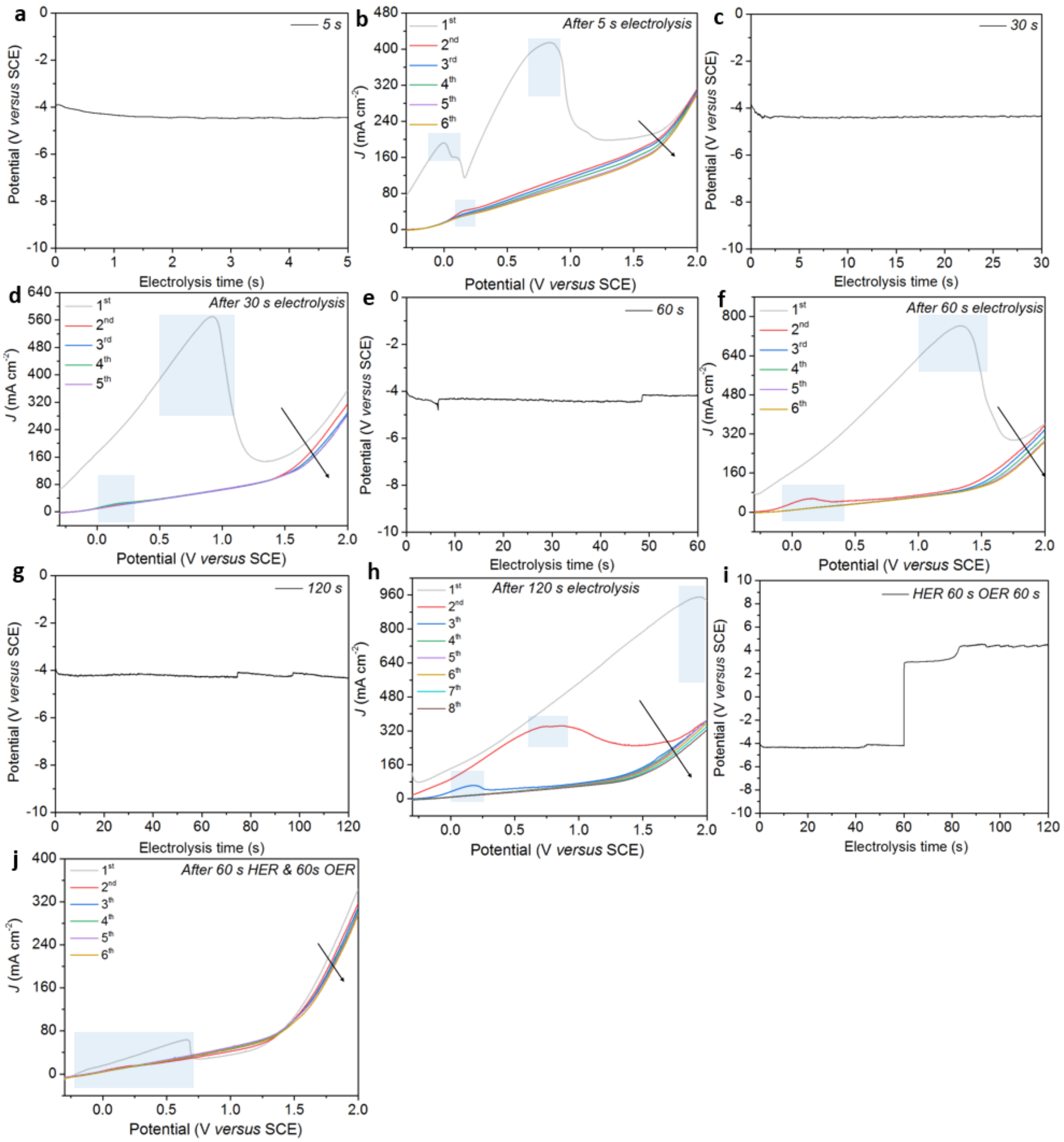

Supplementary Fig. 29 | Chronopotentiometric curves and the corresponding OER curves in 1 M Na^{+} + 0.3 M Co^{2+}. (a) *V*-t curve and (b) polarization curves after 5 s HER electrolysis. (c) *V*-t curve and (d) polarization curves after 30 s HER electrolysis. (e) *V*-t curve and (f) polarization curves after 60 s HER electrolysis. (g) *V*-t curve and (h) polarization curves after 120 s HER electrolysis. (i) *V*-t curve and (j) polarization curves after 60 s HER-60 s OER electrolysis. Supplementary Fig. 29h shows that the surface is not stable at the initial stage and surface species are gradually oxidized completely.

Supplementary Fig. 29j does not show the prominent oxidation peaks for the 1st scan similar to Supplementary Fig. 29b, 29d, 29f, and 29h, probably because the material formed during repair step oxidized at the beginning of the alternating electrolysis OER step, and the complete O_2 evolution began only after the material oxidation was complete.

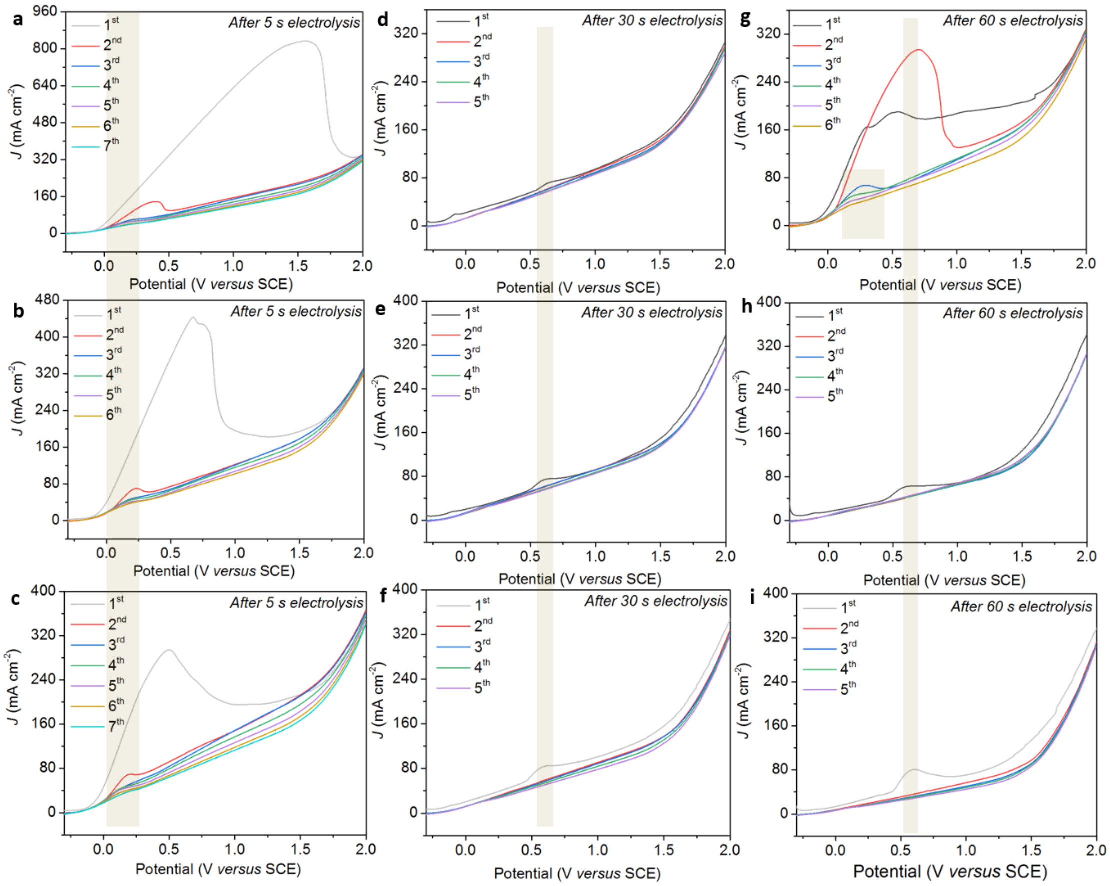

Supplementary Fig. 30 | OER curves after chronopotentiometric tests (5 s, 30 s and 60 s) at fixed *J* (negative potentials). Polarization curves recorded in acidic Co^{2+}-free solutions containing (a) 1.3 M Li^{+}, (b) 1.3 M Na^{+} and (c) 1.3 M K^{+} after 5 s chrono-potentiometric tests. Curves recorded after (d-f) 30 s and (g-i) 60 s chrono-potentiometric tests. The first row of data is collected in solution containing 1.3 M Li^{+}. The second row of data is collected in solution containing 1.3 M Na^{+}. The third row of data is collected in solution containing 1.3 M K^{+}.

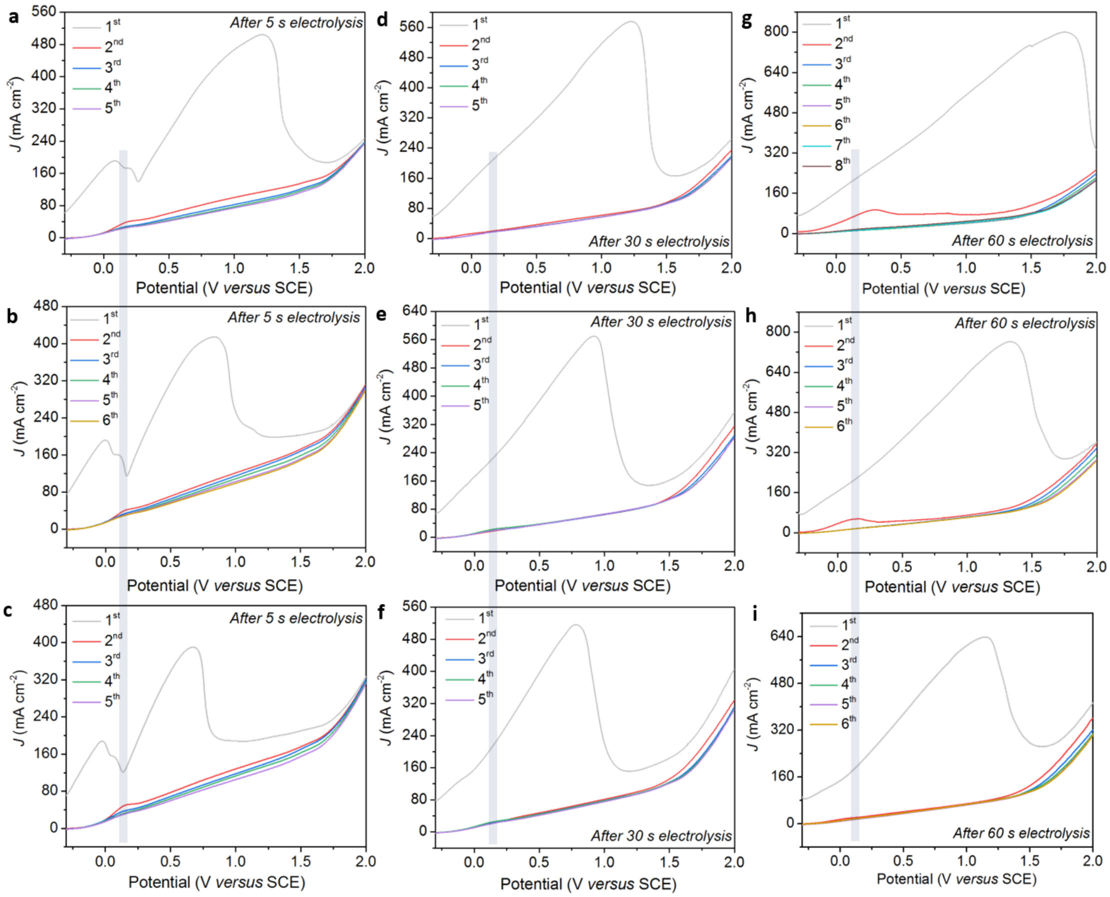

Supplementary Fig. 31 | OER curves after chronopotentiometric tests (5 s, 30 s and 60 s) at fixed *J* (negative potentials). Polarization curves recorded in acidic solutions containing (a) 1 M Li^+ + 0.3 M Co^{2+}, (b) 1 M Na^+ + 0.3 M Co^{2+} and (c) 1 M K^+ + 0.3 M Co^{2+} after 5 s chrono-potentiometric tests. Curves recorded after (d-f) 30 s and (g-i) 60 s chrono-potentiometric tests. The first row of data is collected in solution containing 1 M Li^+ + 0.3 M Co^{2+}. The second row of data is collected in solution containing 1 M Na^+ + 0.3 M Co^{2+}. The third row of data is collected in solution containing 1 M K^+ + 0.3 M Co^{2+}.

The J at 2 V_{SCE} in electrolytes containing Li^+ are generally lower than the J at 2 V_{SCE} recorded in electrolytes containing Na^+ and K^+. From the data in each row, longer the step for the HER is prolonged, more species that can be oxidized are accumulated (for the 1st OER scan). Huge oxidation peaks rarely appear after 30 s and 60 s in electrolytes without Co^{2+} (Supplementary Fig. 30). While such oxidation peaks are still present after 30 s and 60 s in electrolytes containing Co^{2+}. LSV curves of OER after 5 s, 30 s, and 60 s HER steps show that the peaks between 0 V_{SCE} and 0.25 V_{SCE} gradually decrease after each scan. And in the presence of 0.3 M Co^{2+}, the position of oxidation peaks are basically between 0 V_{SCE} and 0.5 V_{SCE} after the 1st scan (unlike the Supplementary Fig. 30d–f).

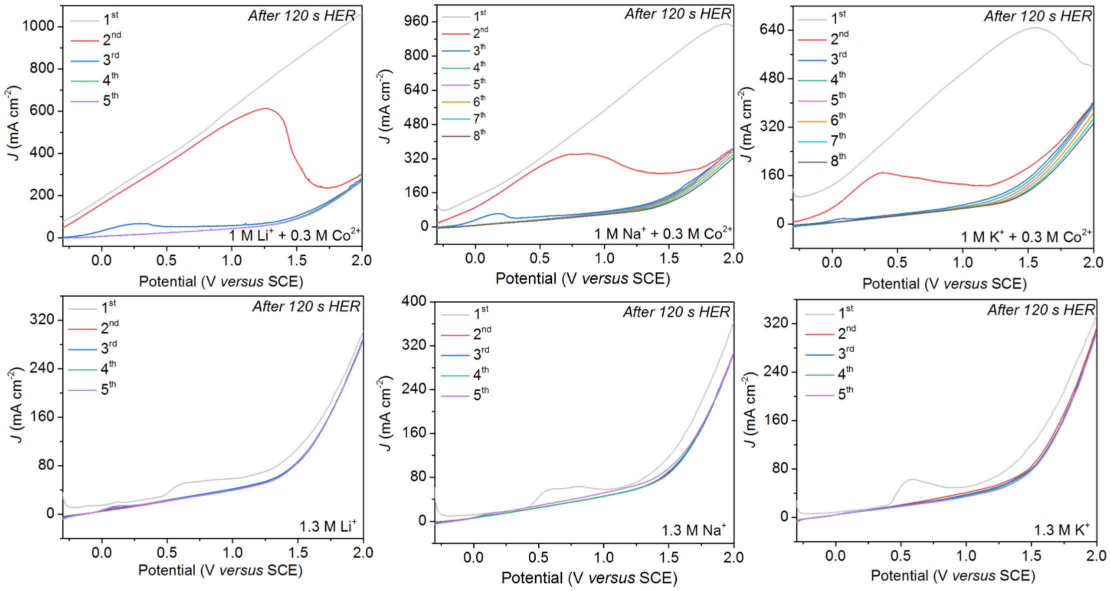

Supplementary Fig. 32 | OER curves after chronopotentiometric tests at fixed *J* (at negative potentials). After the 120 s HER step, oxidation curves in solution containing Co^{2+} are different from those in the Co^{2+}-free solution.

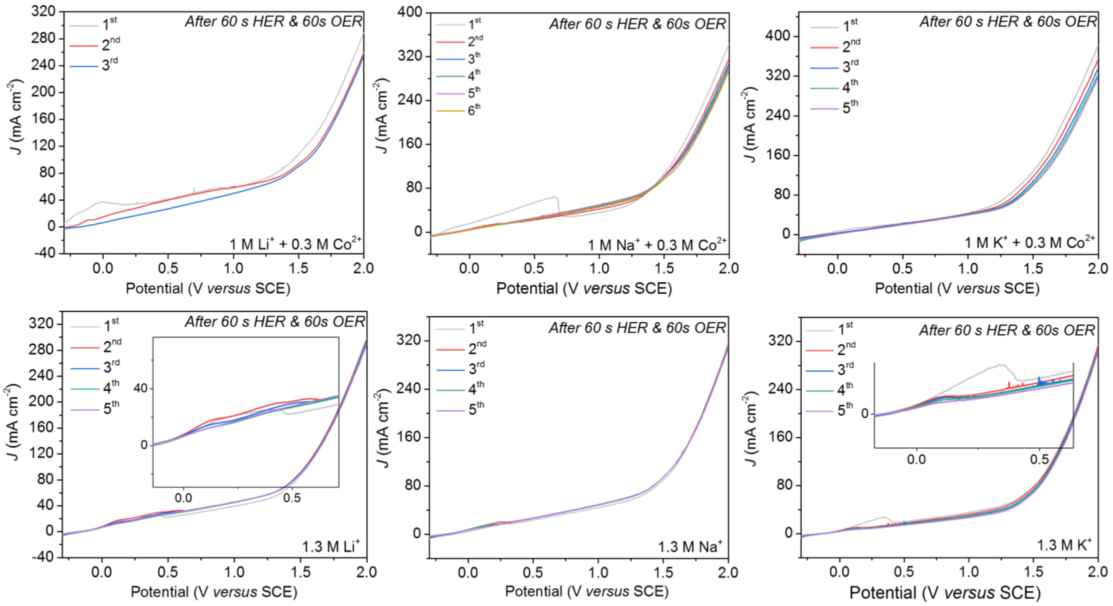

Supplementary Fig. 33 | OER curves after alternating electrolysis tests at two fixed *J* (60 s at negative potentials and 60 s at positive negative potentials). After the 60 s HER step and subsequent 60 s oxidation, less species can be oxidized on the electrode surface. By comparing data in the 2nd row of Supplementary Figs. 31 and 32, different scanning modes bring different OER curve features.

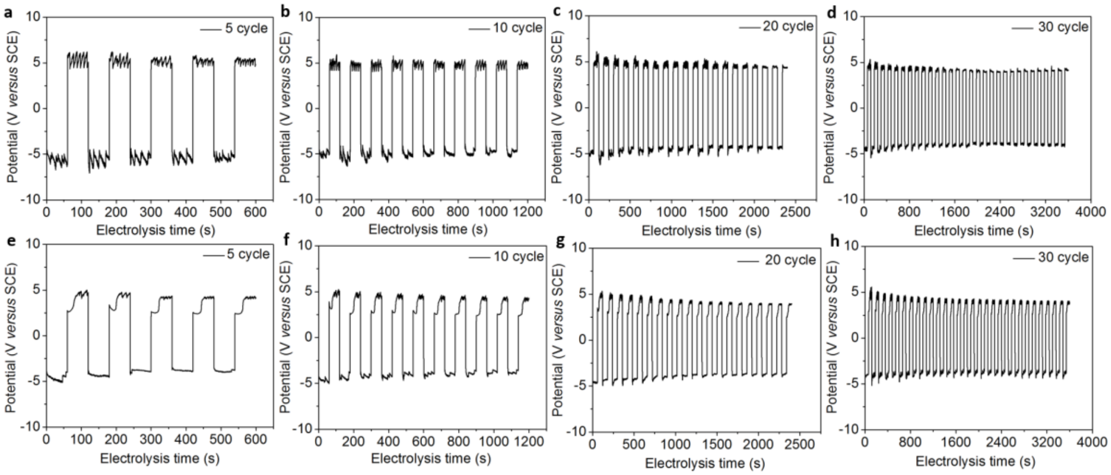

Supplementary Fig. 34 | *V*-t curves of alternating electrolysis at two fixed *J* (60 s at negative potentials and 60 s at positive negative potentials) in different solutions. Alternating electrolysis plots recorded on NiFE in (a-d) acidic solutions with 1.3 M Na^+ and (e-h) with 1 M Na^+ + 0.3 M Co^{2+}.

The jagged curve fluctuations are caused by the rapid release of gas bubbles from the electrode surface. The alternating electrolysis based on the co-action of Na^+ and Co^{2+} alters the behavior of the electrode potential changes recorded in the solution containing only Na^+. According to the *V*-t curves, changes occurs in both two stages under negative or positive potentials, and more significant changes are observed in the OER stage under positive potentials.

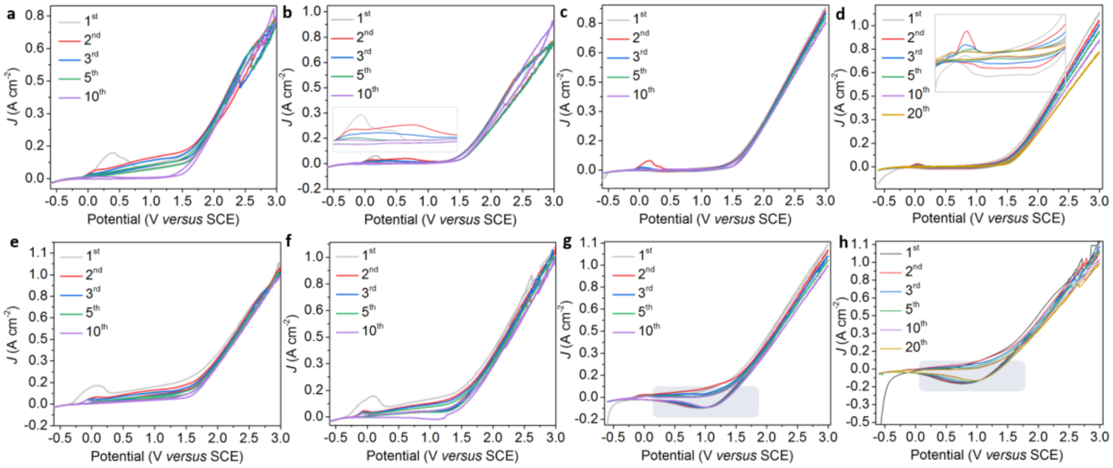

Supplementary Fig. 35 | CV curves recorded after different number of alternating electrolytic cycles (a cycle consisting of a period of HER and a subsequent period of OER). (a) CV curves recorded on NiFE in 1.3 M Na^{+}. (b) CV curves recorded on NiFE in 1 M Na^{+} + 0.3 M Co^{2+}.

Eight separate pieces of NiFE are used in each of the eight Figures. Each row from left to right is the CV curves recorded after different alternating electrolysis cycles (5 cycles, 10 cycles, 20 cycles, and 30 cycles). Generally, the oxidation peak area decreases with the increase of the alternating electrolysis cycles (from 5 to 30 cycles). Noticeably, oxidation peaks negatively shift in the presence of Co^{2+}, thus representing distinct in situ generated species. Additionally, the emergence of irreversible reduction peaks after extended cycles (e.g., 20 cycles, and 30 cycles) implies that some high-valent Co species are created and retained on the surface the NiFE after the alternating steps (i.e., negative potential, positive potential, negative potential, positive potential, negative potential, …).

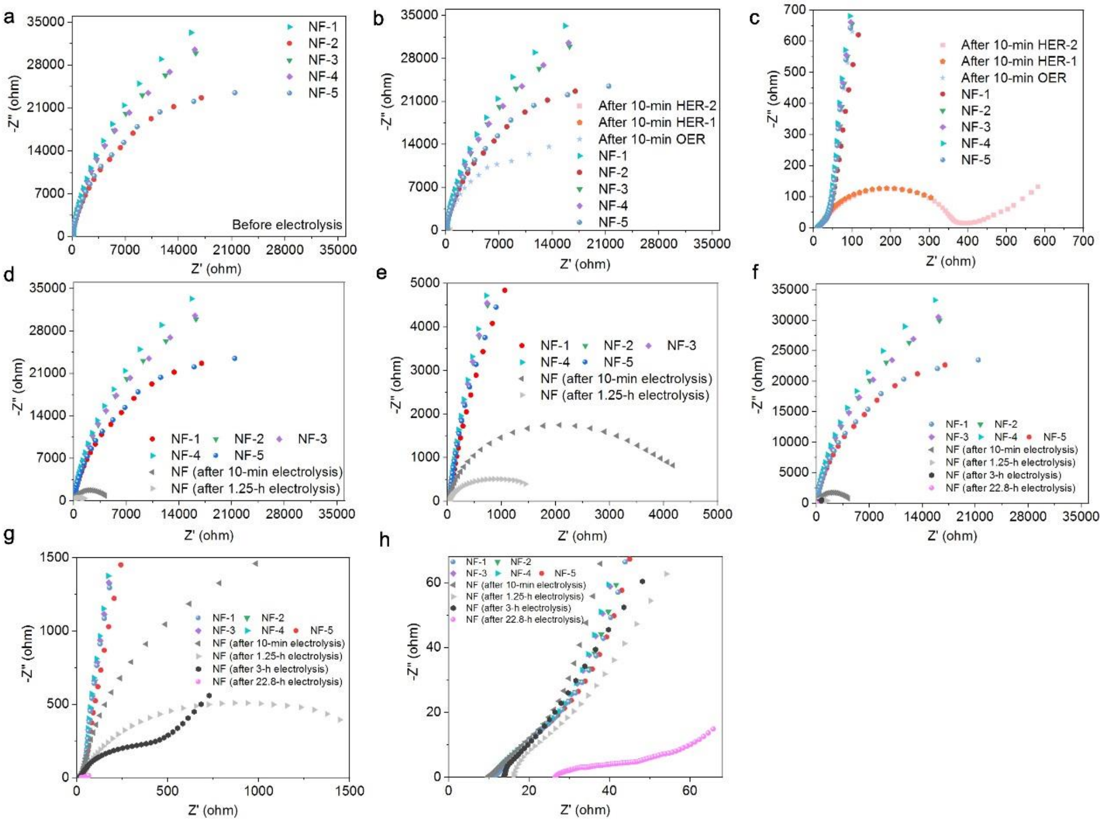

Supplementary Fig. 36 | Detailed EIS data comparison. (a) Independent EIS data of five pieces of Ni foam (NF) electrodes before and after testing in different acidic H_2SO_4 solution with different ions, including NF-1, NF-2, NF-3, NF-4, and NF-5. (b) Comparison of the EIS data before electrolysis and after conventional OER/HER electrolysis. Tests for the HER employed different low-frequency parameters, so there are two impedance results (the impedance arc is basically the same). (c) Magnification of details. (d) Comparison of the EIS data before electrolysis and after AE for 10 min and 12.5 h. (e) Magnification of details. (f) Comparison of the EIS data before electrolysis and after AE for 10 min, 12.5 h, 3 h, and 22.8 h. (g) Magnification of details. (h) Magnification of details.

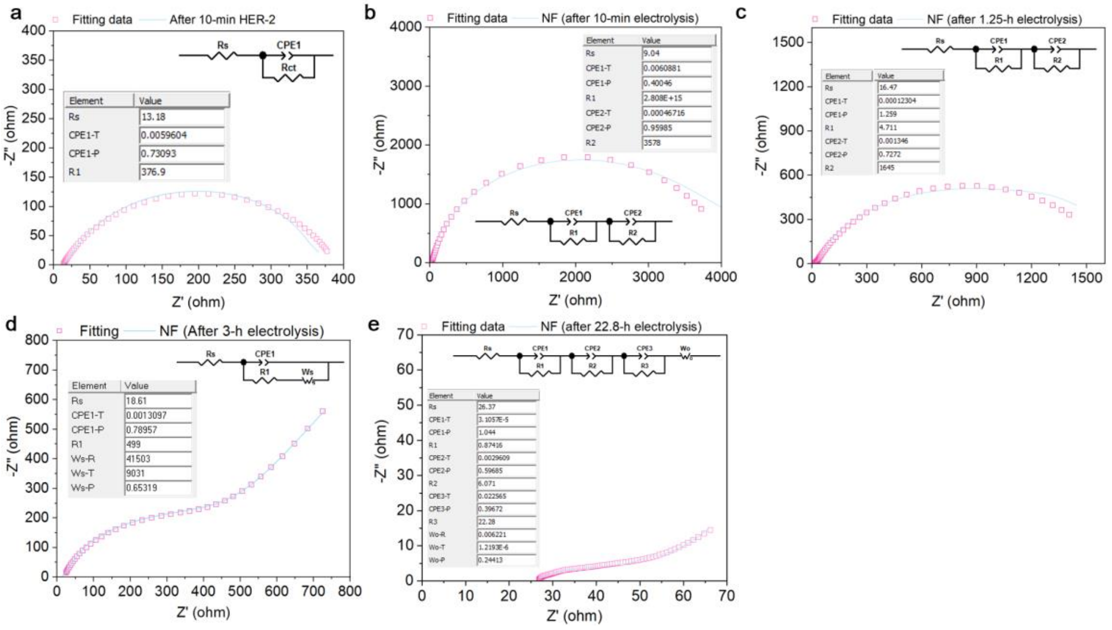

Supplementary Fig. 37 | EIS fitting results. (a) EIS fitting results for sample after 10-min HER testing. (b) EIS fitting results for sample after 10 min of AE testing. (c) EIS fitting results for sample after 1.25-h AE testing. (d) EIS fitting results for sample after 3-h AE testing. (e) EIS fitting results for sample after 22.8-h AE testing. The equivalent circuit used for modeling the measured electrochemical response are given as inset images. CPE stands for constant phase angle element. Rct, R1, R2, and R3 are related with the kinetics of the interfacial charge transfer reaction. Rs represents the solution resistance (*J. Am. Chem. Soc.* **142**, 12087–12095 (2020), *Nat. Commun.* **14**, 1873 (2023), *Electrochim.* Acta **418**, 140350 (2022)).

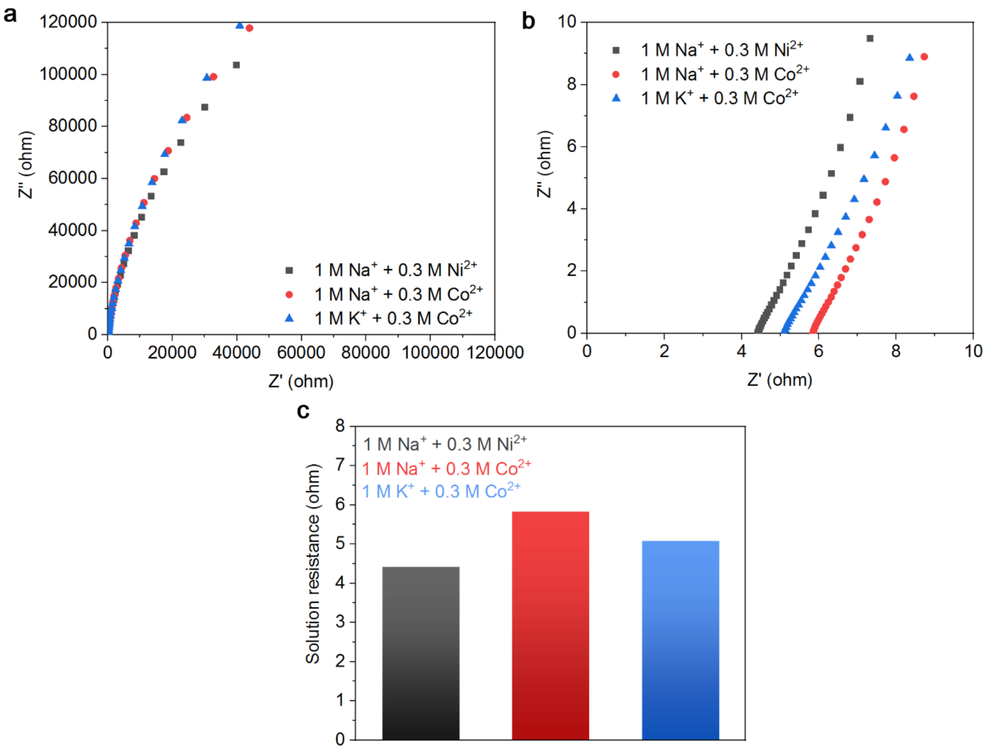

Supplementary Fig. 38 | Comparison of resistance values. (a) Comparison of Nyquist plots in different electrolytes. (b) Magnification of details. (c) Comparison of the solution resistance values. All the three independent tests used the same the volume of electrolyte, the same working electrode, the same reference electrode, and the same counter electrode. The only variable was the ionic species of the electrolyte.

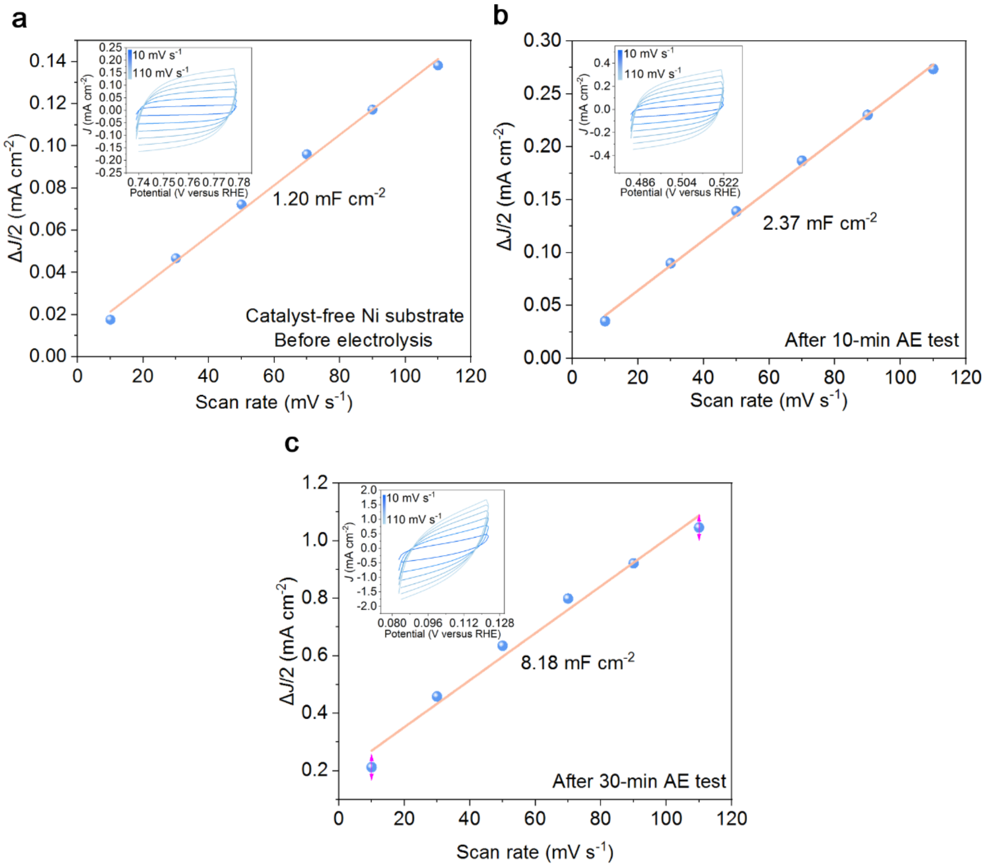

Supplementary Fig. 39 | Changes in the C_{dl} value. (a) C_{dl} value of Ni foam before electrolysis. The inset shows the corresponding cyclic voltammograms at scan rates of 10, 30, 50, 70, 90, and 110 mV s^{-1}. (b) C_{dl} value of Ni foam after a 10-min AE process and the related cyclic voltammograms. (c) C_{dl} value of Ni foam after a 30-min AE process and the related cyclic voltammograms. All electrochemical tests were recorded in 0.5 M Na_2SO_4 aqueous electrolyte.

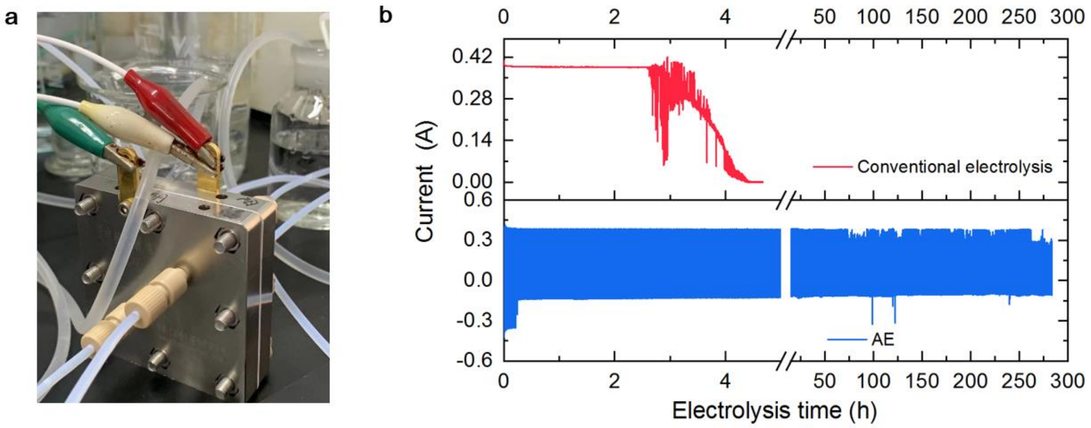

Supplementary Fig. 40 | PEM-based cell tests under room temperatures. (a) Photo of the assembled PEM water electrolyzer. (b) Comparison of electrolysis time length under two different conditions. The applied cell voltage is 2.8 V. The electrochemical tests were recorded in aqueous electrolyte with 0.2 M Na^{+} (an excess content) and 60 mM Co^{2+}.

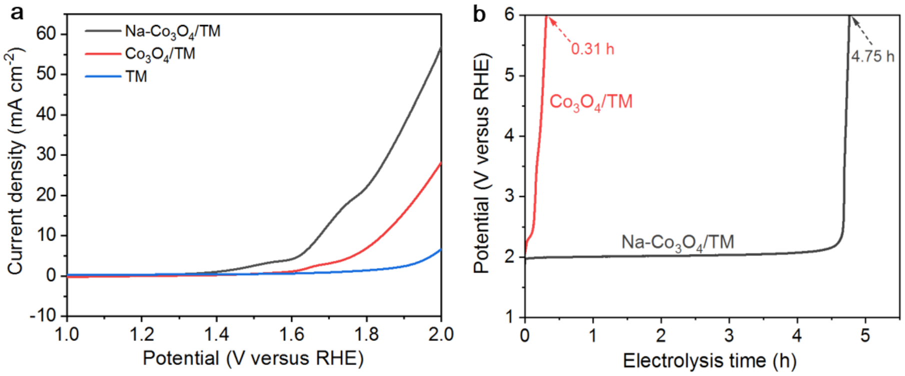

Supplementary Fig. 41 | Comparison of the performance of catalysts with two different synthesis schemes. (a) Polarization curves of different electrodes in H_2SO_4 solution (pH = 1). (b) Stability comparison of Co_3O_4/TM and Na-Co_3O_4/TM.

We have coated Ti mesh with Co_3O_4 (Co_3O_4/TM) and Ti mesh with Na-doped Co_3O_4 (Na-Co_3O_4/TM) according to a previous work (*Appl. Catal. B* **317**, 121769 (2022)) and studied the stability performance in acidic media. Interestingly, even the direct Na doping enhances the stability of Co_3O_4, with results showing that the Na-Co_3O_4/TM achieves 15.3 times the electrolysis time of the Co_3O_4/TM (4.75 h versus 0.31 h). This result further affirms the significance of our work, i.e., alkali metals and iron group metals may not only synergize during the alternating electrolysis tests in our present work, but better electrocatalytic performance in acidic solution may also be achieved by preparing them directly as the electrocatalysts.

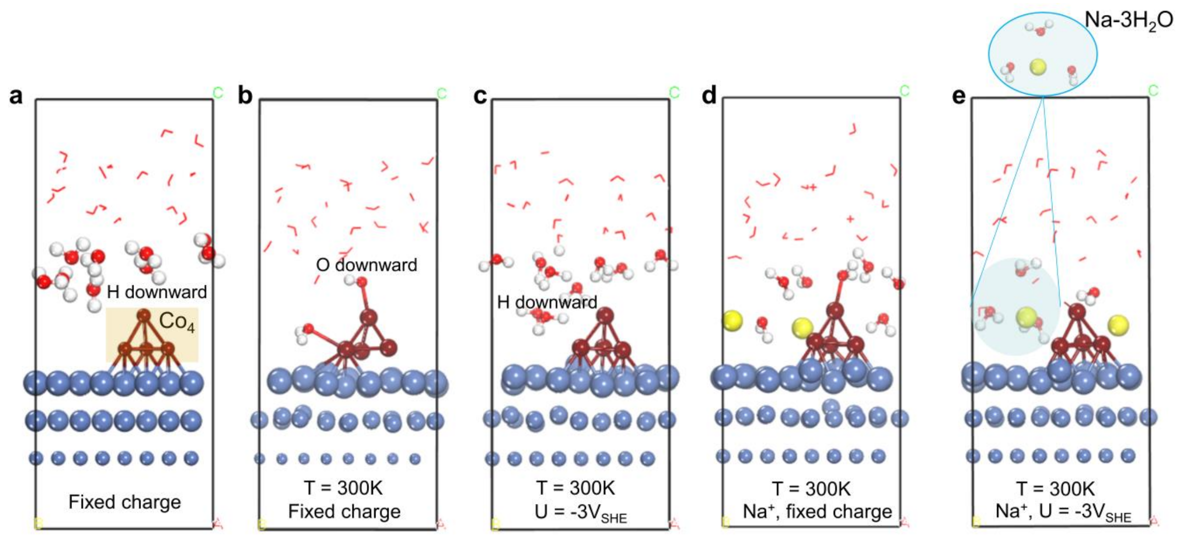

Supplementary Fig. 42 | Comparison of surface reaction processes at different simulation conditions. (a-e) Catalyst-electrolyte interfaces with/without AMCs, respectively.

To verify the effectiveness of the CPMD approach, we first test the traditional "static" DFT calculation without considering electrode potential (fixed charge), see Supplementary Fig. 42a. The H atoms in the water molecules move toward the Ni (111) catalyst, while the Co-containing layer is adsorbed on the surface of Ni (111) without interacting chemically with the water. Then, we use the MD calculation without considering electrode potential to examine the interface behavior under dynamic conditions, see Supplementary Fig. 42b. Two water molecules are spontaneously adsorbed on the Co-containing layer, and they are respectively adsorbed on two Co atoms, making the adsorbed Co atoms away from the surface. The metal Co is easily oxidized by oxygen atoms in water. The comparison of static and dynamic calculations shows two completely different interface structures. Among them, the dynamic simulation is safer because it is closer to the real environment[64], and thus is conducted in our subsequent electrochemical interface investigations.

When the potential is set to $-3V_{SHE}$ in the dynamic CPMD simulation in Supplementary Fig. 42c, the adsorbed H_2O molecules spontaneously leave the Co-containing layer, returning it to the lower valence states. It results from the applied negative potential forming a reducing interface environment. Until here, it can be firmly concluded that besides considering explicit water solvent, the consideration of both real electrode potential (U) and molecular dynamics (T) is necessary, as they affect changes in the solvent, adsorbed species, and catalyst structure.

Then, we added Na^+ into the interface and found that using MD calculations without considering electrode potential, Na^+ does not form a hydrated state with water, see Supplementary Fig. 42d. This phenomenon violates the actual mechanism by which Na^+ is generally hydrated in water[65]. Interestingly, when we perform CPMD calculation using $U = -3\ V_{SHE}$, Na^+ forms a hydrated state with three water molecules, see Supplementary Fig. 42e. According to the above findings, it can be said that the state-of-the-art CPMD approach can simulate the electrocatalyst/electrolyte interface more accurately.

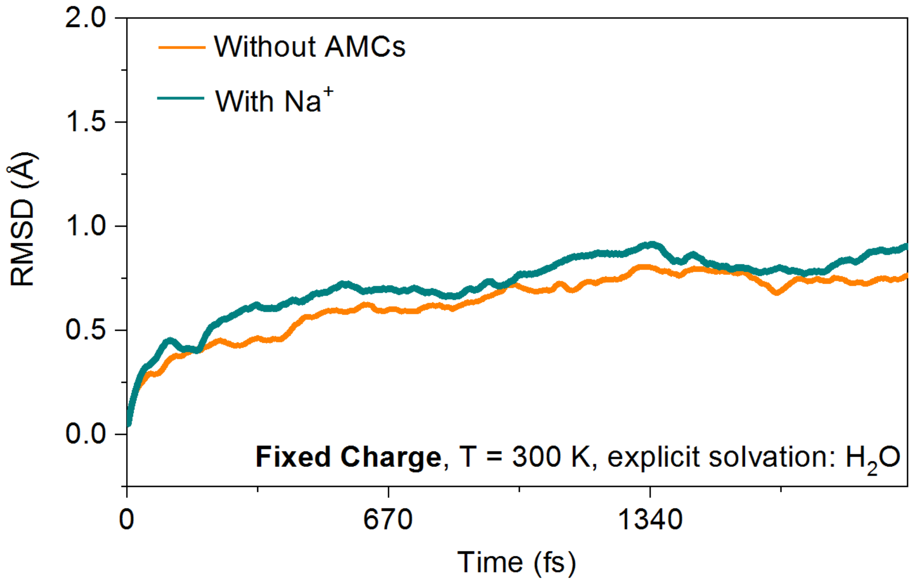

Supplementary Fig. 43 | RMSD with Na^+ and without AMCs at a fixed charge (without considering electrode potential)

Calculation results show that using MD calculations without considering electrode potential, there is no significant difference between the RMSD with and without Na^+. It suggests that the consideration of real electrode potential (U) in simulation is necessary.

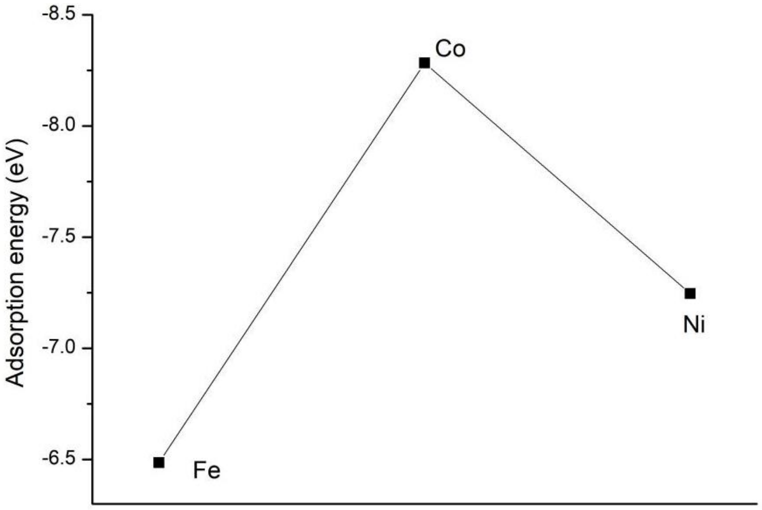

Supplementary Fig. 44 | Adsorption energies (Ea) of Fe, Co and Ni atoms on Ni (111).

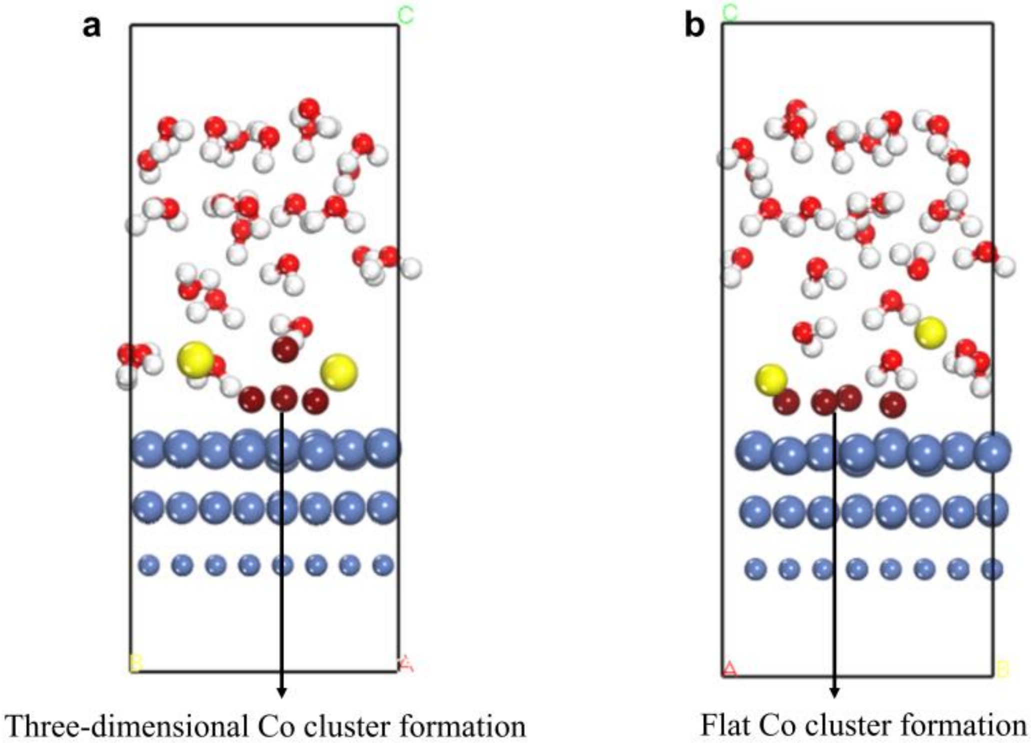

Supplementary Fig. 45 | Three-dimensional (a) and flat (b) Co cluster formation on Ni (111).

We investigated the three-dimensional and flat Co cluster formation on the Ni (111) by using grand canonical ensemble of electron (GCE), see Supplementary Fig. 45. Note that the flat Co cluster formation represents the growth of uniform protective coating on the Ni (111) and vice versa. Our calculation results show that the flat Co cluster forms with a formation energy (E_f) of 1.07 eV, which is lower than that three-dimensional Co cluster formation with E_f = 1.26 eV. The Co metal prefers more to deposit uniformly on the Ni core, which is consistent with the experiment observation.

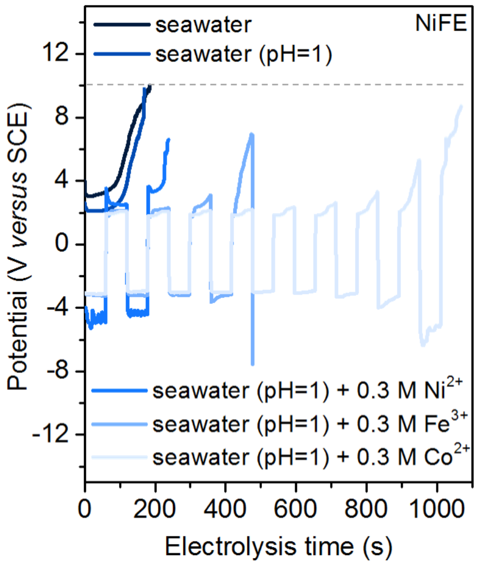

Supplementary Fig. 46 | Lifespan of NiFE under different conditions (*V*-t curve in seawater, *V*-t curve in acidified seawater, and alternating electrolysis curves with different metal ions). Bare NiFE is highly unstable during natural seawater electrolysis, and its dissolution rate is too fast.

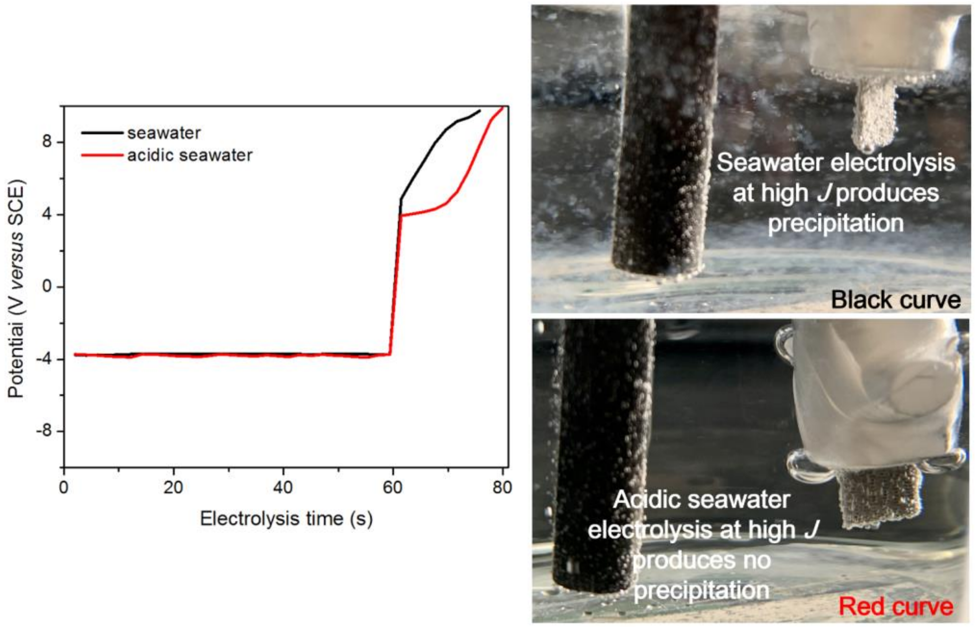

Supplementary Fig. 47 | Alternating electrolysis using bare TiME under the fixed *J* of 1 A cm^{-2} and −1 A cm^{-2}.

Since the Ti mesh substrate does not have much OER activity, the anions including Cl^- and OH^-, SO_4^{2-}, CO_4^{2-}, *etc*., accumulated on the anode surface will not be consumed in time after adsorption, resulting in electrolysis failure even with brine water.

Note that the majority of reported seawater electrolysis works used alkaline seawater, while electrolysis in acidic seawater has the following benefits:

1) The production of unwanted Ca/Mg precipitates does not occur in acidic seawater. Note that Ca/Mg precipitates would generate at a high rate during natural seawater reduction. Alkaline seawater is obtained by adding expensive KOH to the seawater prior to electrolysis and then pre-filtering the Ca/Mg precipitates.
2) Electrolysis in acidic seawater will facilitate the kinetics of HER.
3) Acidic seawater electrolysis favors the production of Cl_2 rather than O_2.

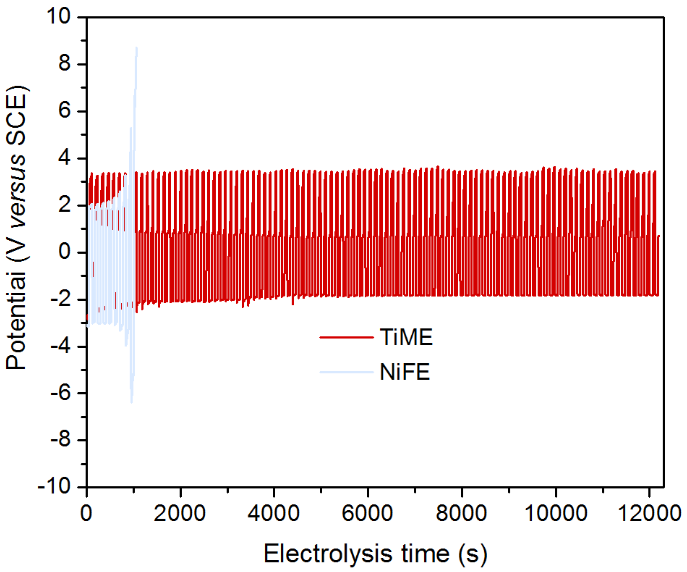

Supplementary Fig. 48 | Alternating electrolysis using different substrates (Ti mesh and Ni foam) under almost identical acidic conditions (0.3 M Co^{2+}, *J* are fixed at 2 A cm^{-2} and −2 A cm^{-2}). Additional 0.5 M Na_2SO_4 is added to reduce the potential required for the TiME to reach a certain *J* so that the reaction can proceed smoothly.

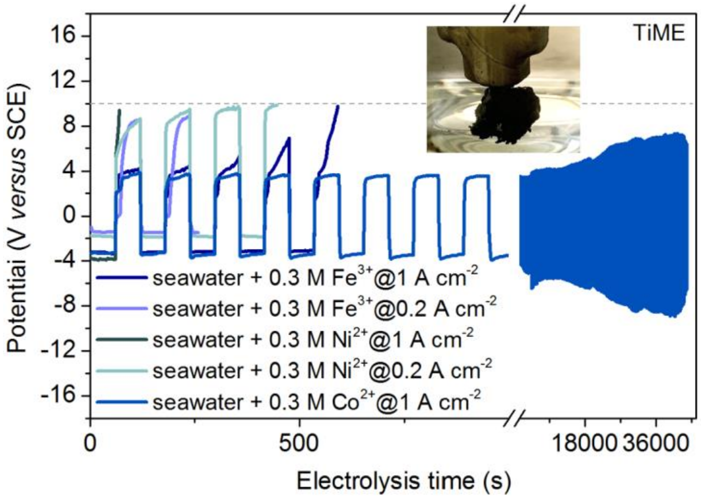

Supplementary Fig. 49 | Alternating electrolysis under different conditions, with various ions. The inset shows that the surface coating of Ti electrode is excessive after electrolysis in seawater with Co^{2+}. The deposition rates are higher than dissolution rates in neutral natural seawater, leading to excessive thickness of coating and gradual decay of activity.

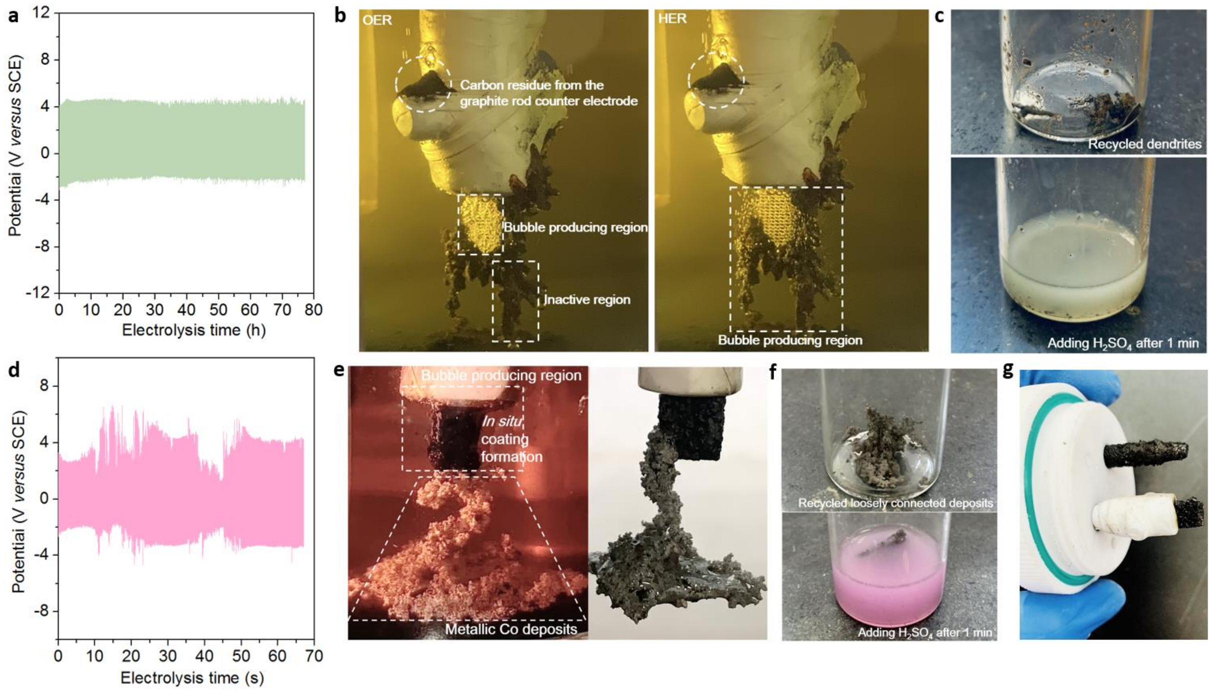

Supplementary Fig. 50 | Long time seawater electrolysis under different conditions and the recovery of waste (fixed *J*: of −1 & 1 A cm^{-2}). (a) Alternating electrolysis *V*-*t* curves based on the Fe^{3+}-Ni^{2+} combo. (b) Photos of the TiME at negative and positive potentials after electrolysis for about 50.4 h (Precipitates grows around the electrode during electrolysis). (c) Fast dissolution of dendrites containing iron and nickel in concentrated sulfuric acid. After electrolysis, the precipitates can be collected and dissolved in acid solution for recycling use. (d) Alternating electrolysis based on the Co^{2+}-Na^{+} combo (Na^{+} comes from the seawater). (e) The TiME surface is evenly covered with a layer of black substance, and the deposit below is mainly metallic Co which was not firmly attached to the Ti substrate in the early stage of electrolysis. Loose parts of the deposits during electrolysis are fall off and build up before they can be oxidized (shot around 51 h). Note that the construction of loose parts does not change significantly after electrolysis. (f) Dissolving such a deposit in acid, changing it back to ionic Co, and then diluting it with seawater for reuse. (g) Obvious black coating on the surface of counter electrode and working electrode. Like the TiME, the counter electrode surface forms a black protective coating, suggesting a large number of Na^{+} in seawater and additional Co^{2+} facilitate the generation of this coating.

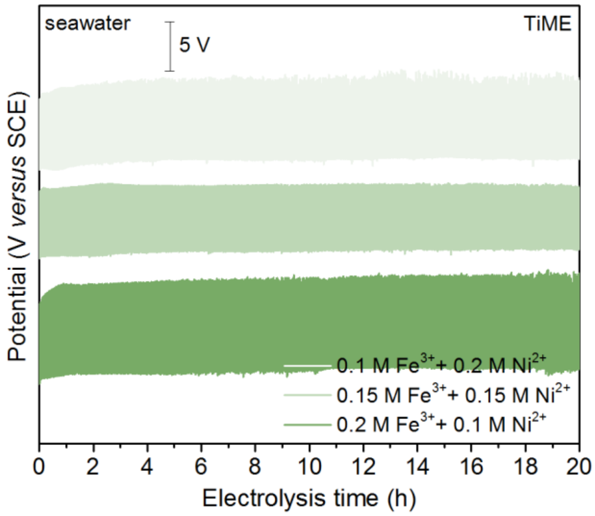

Supplementary Fig. 51 | Long time alternating electrolysis (the *J* are fixed at 1 A cm^{-2} and −1 A cm^{-2}) in seawater containing with different Ni^{2+}-to-Fe^{3+} ratios.

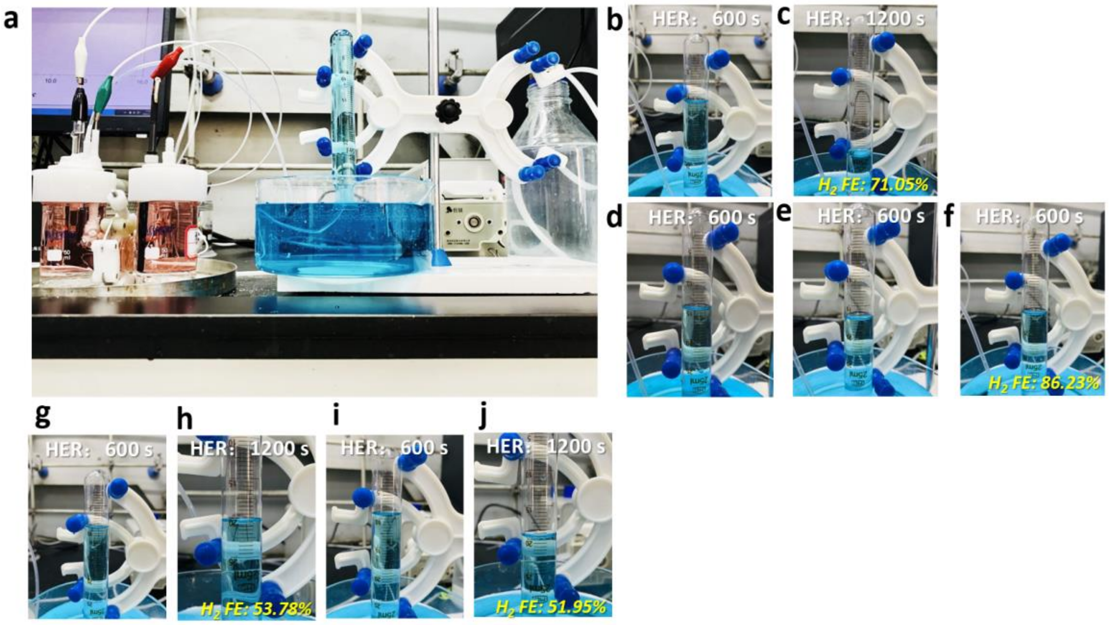

Supplementary Fig. 52 | Tests to determine the FE of H_2 gas under different electrolysis conditions. (a) H_2 production in the HER stage is measured by drainage method. (b,c) H_2 gas accumulation after 600 s of electrolysis when Co^{2+} concentration in the electrolyte is 0.03 M. When the electrolysis time for the HER stage reaches 1200 s, the FE of H_2 is 71.05%. (d-f) H_2 gas accumulation after 600 s of electrolysis in solution with 0.03 M Co^{2+} and 0.1 M Na^+. FEs values exceed 80% in all three tests. (g,h) H_2 gas accumulation after 600 s and 1200 s of electrolysis in solution with 0.09 M Co^{2+}. (i,j) H_2 gas accumulation after 600 s and 1200 s of electrolysis in solution with 0.09 M Co^{2+} and 0.3 M Na^+. Noticeably, the reduced ion concentrations does not significantly affect the electrolysis time that can be achieved, as the corresponding electrolyte acidity can be adjusted.

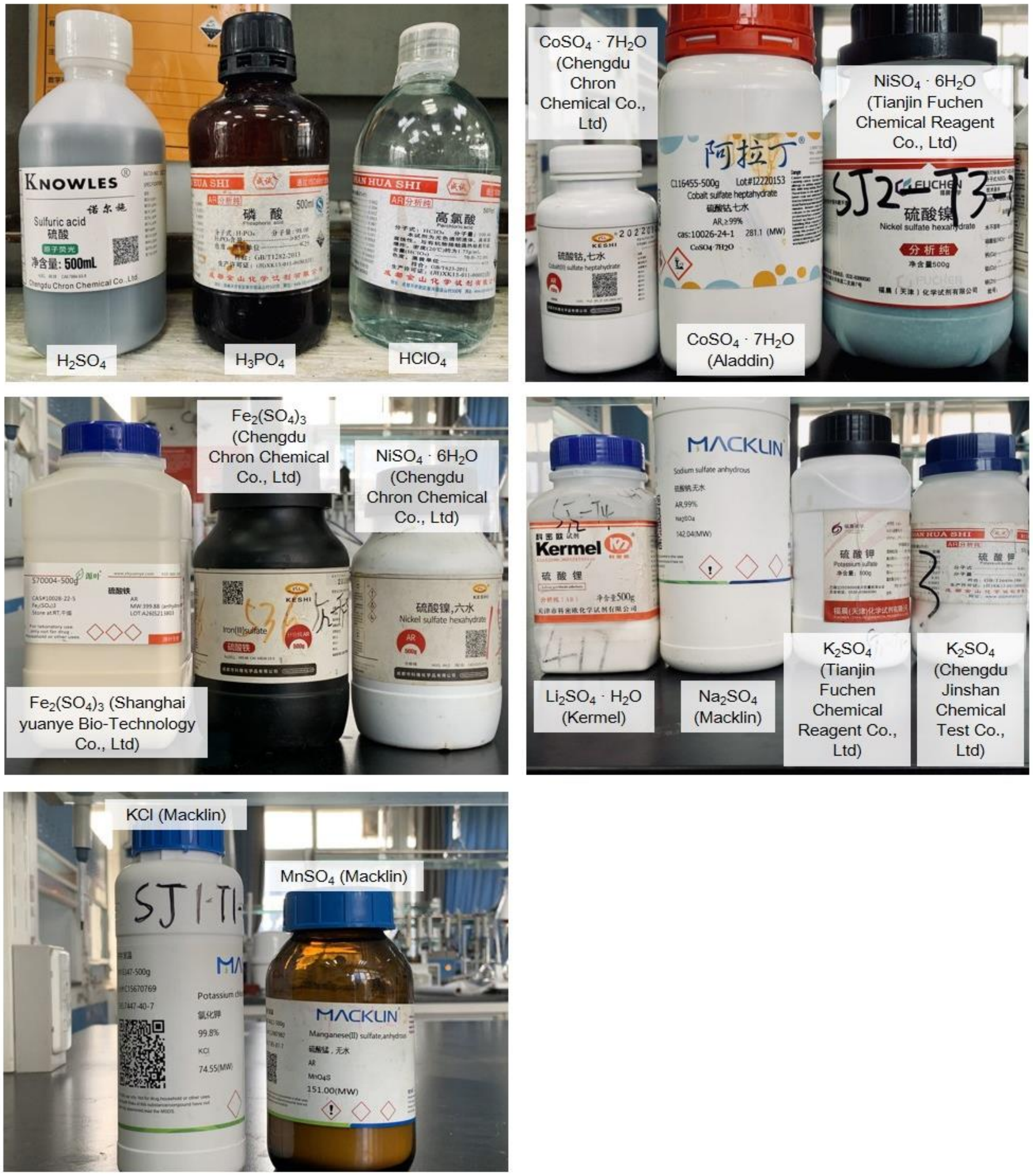

Supplementary Fig. 53 | Photos of the main chemical reagents used for the experiments in this work.

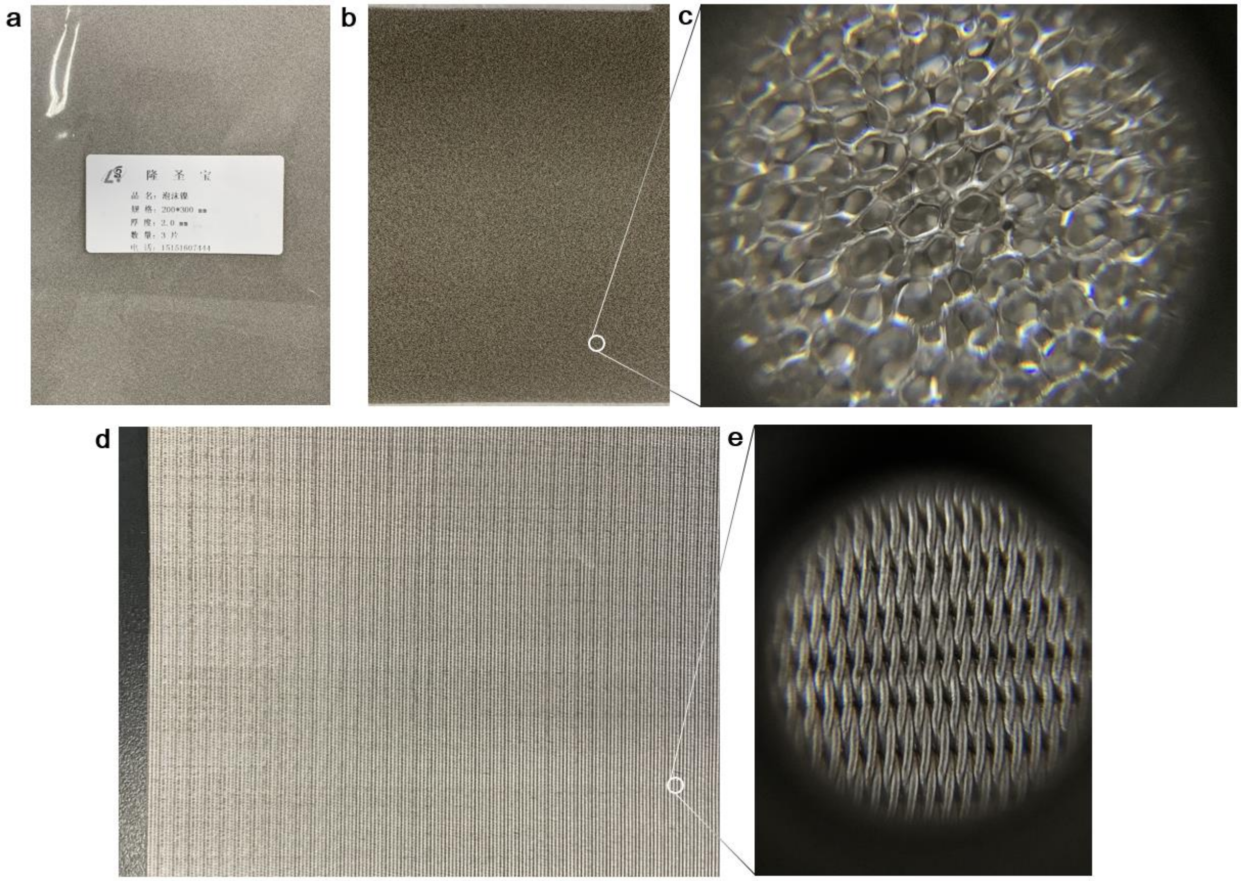

Supplementary Fig. 54 | Photos of the metal substrates used in this work. (a,b) Photos of the nickel foam and (c) the corresponding enlarged image providing more details. (d) Photo of the titanium mesh and the corresponding (e) enlarged image. Enlarged images of nickel foam and copper foam are not included with scale bars because they were taken via common magnifying lens.

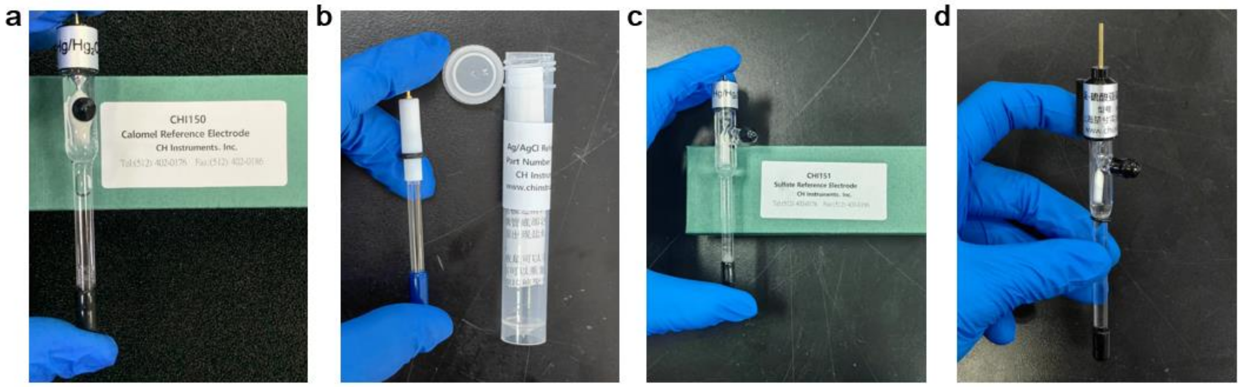

Supplementary Fig. 55 | Photos of the different reference electrodes. (a) Saturated calomel electrode purchased from CH Instruments Ins (the website: http://www.chinstr.com/sy). (b) Ag/AgCl electrode purchased from CH Instruments Ins. (c) Sulfate reference electrode purchased from CH Instruments Ins. (d) Sulfate reference electrode purchased from SHANGHAI CHUXI INDUSTRIAL CO., LTD (the website: http://www.chuxi17.com/).

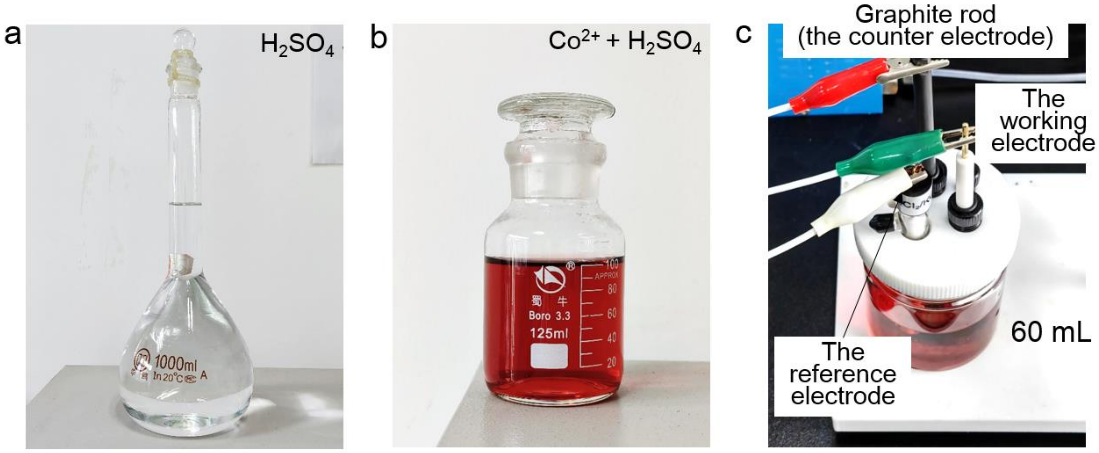

Supplementary Fig. 56 | Simplified processes for preparing electrolyte and single-chamber cell. (a) Photos of the acidic H_2SO_4 solution for preparing the electrolyte. (b) Photos of the acidic solution with Na^+ and Co^{2+}. (c) Undivided electrolyzer for screening ion combinations.

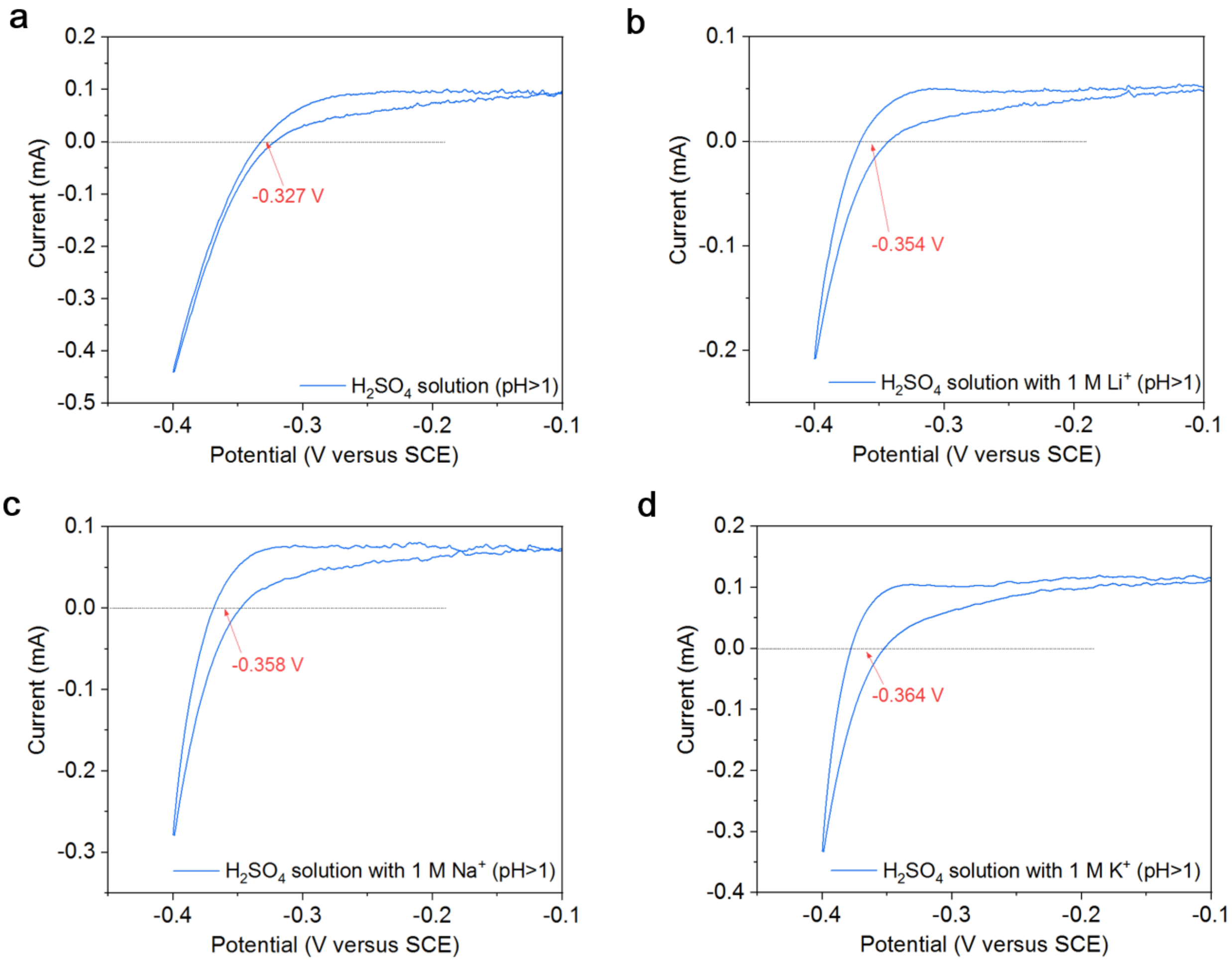

Supplementary Fig. 57 Voltammetry curves for the calibration of Hg/Hg_2Cl_2 electrodes in (a) H_2SO_4 solution, (b) H_2SO_4 solution with 1 M Li^+, (c) H_2SO_4 solution with 1 M Na^+, and (d) H_2SO_4 solution with 1 M K^+ (solution pH > 1).

Experimental procedures for correcting the reference electrodes can be find in previous literature (e.g., *ACS Energy Lett.* **5**, 1083–1087 (2020), *ACS Catal.* **13**, 1893–1898 (2023), and *Nat. Mater.* **10**, 780–786 (2011)). The fluctuations in the curves are probably due to the intense H_2 bubble flow (Supplementary Fig. 58b). The limited geometry surface area and the limited active sites of the Pt plate should be the reasons for the small currents of the polarization curves. Besides, we used the RHE calibration data of Hg/Hg_2Cl_2 electrode obtained in solutions without the addition of iron group element ions to calibrate the data for all solutions with the addition of iron group element ions because (i) the difference in pH value is not significant and (ii) the metal ions may be deposited in the potential range of the CV tests.

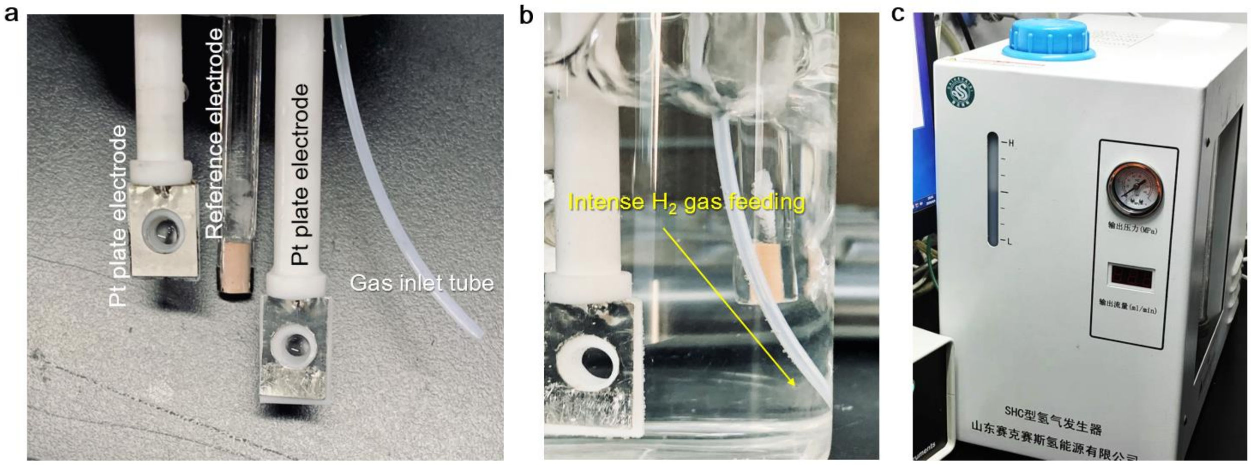

Supplementary Fig. 58 (a) Photo of the Pt plate electrodes, a gas tube, and a reference electrode. (b) Rapid release of H_2 bubbles from the gas tube during correction tests. (c) Hydrogen generator used in this work.

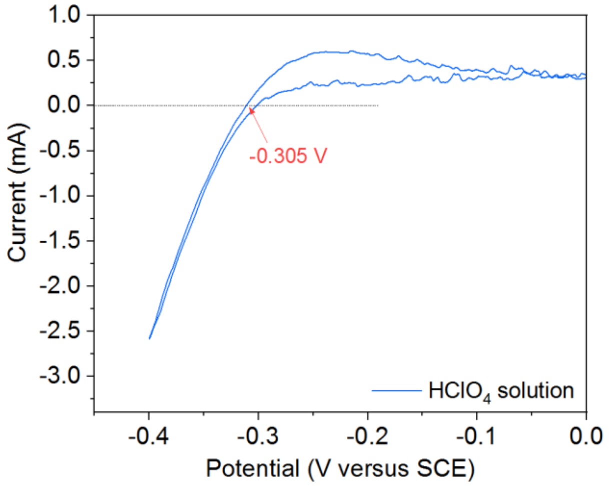

Supplementary Fig. 59 Voltammetry curve for the calibration of Hg/Hg_2Cl_2 electrodes in 0.1 M $HClO_4$ solution. To provide more information of the Hg/Hg_2Cl_2 electrode, we also provide the calibration data in $HClO_4$ solution of known a concentration in that $HClO_4$ ionizes 100% in solution.

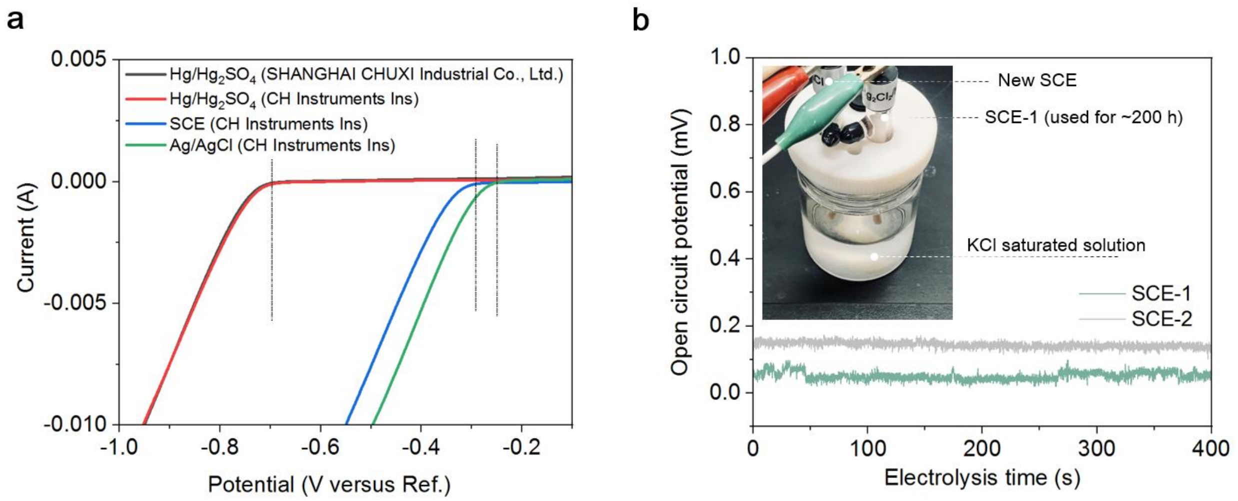

Supplementary Fig. 60 (a) Polarization curves of a commercial Pt plate electrode recorded with different reference electrodes in the same acidic electrolyte. The potentials are voltages recorded directly by the electrochemical workstation. (b) Time-dependent open-circuit potential values.

For the measurements in Supplementary Fig. 60b, connecting a new SCE electrode to the reference electrode clamp and counter electrode clamp, and connecting the SCE electrodes after hundreds of hours of use to the working electrode clamp. SCE-1 was used for about 200 h and SCE-2 was used for about 150 h. The tests further indicate that the used SCE electrodes were not damaged.

Supplementary Table 1. Lifespan comparison of our catalytic systems with selected catalysts designed for the operation at low pH (< 3).

Catalyst/support	Lifespan (h@mA $cm_{geo.}^{-2}$)	pH value	Ref.
NaCo-based coating/NiFE	93.8 h@2000 mA cm^{-2}&−2000 mA cm^{-2}	2>pH>1 H_2SO_4	This work
NiFE	2 h@1000 mA cm^{-2}	2>pH>1 H_2SO_4	This work
Co_2MnO_4/Pt/Ti mesh Co_2MnO_4/FTO	1500 h@200 mA cm^{-2} (from Fig. 3b) 1400 h@200 mA cm^{-2} (from Fig. 3b)	pH=1 H_3PO_4	*Nat. Catal.* **5**, 109–118, (2022)
Co_2MnO_4/FTO	~3 h@1000 mA cm^{-2} (from Fig. 3b) ~7 h@1000 mA cm^{-2} (from Fig. 3b)	pH=1 H_2SO_4 pH=1 H_3PO_4	*Nat. Catal.* **5**, 109–118, (2022)
Ni-RuO_2/platinized Ti fibre felt electrode (high pressure pressed)	1000 h@200 mA cm^{-2} (from Fig. 5f)	0.1 M $HClO_4$	*Nat. Mater.* **22**, 100–108, (2023)
Ru_1–Pt_3Cu/glassy carbon electrode	28 h@10 mA cm^{-2} (from Fig. 3c)	0.1 M $HClO_4$	*Nat. Catal.* **2**, 304–313 (2019)
12Ru/MnO_2/carbon cloth	200 h@10 mA cm^{-2} (from Fig. 3d)	0.1 M $HClO_4$	*Nat. Catal.* **4**, 1012–1023 (2021)
GB-$Ta_{0.1}Tm_{0.1}Ir_{0.8}O_{2-\delta}$/Au disc electrode	500 h@10 mA cm^{-2} (from Fig. 3d)	0.5 M H_2SO_4	*Nat. Nanotechnol.* **16**, 1371–1377 (2021)
GB-$Ta_{0.1}Tm_{0.1}Ir_{0.8}O_{2-\delta}$/PTFE (Pt-plated Ti foam acts as anode gas diffusion layer)	500 h@1500 mA cm^{-2} (from Fig. 3d)	0.5 M H_2SO_4	*Nat. Nanotechnol.* **16**, 1371–1377 (2021)
$Ir_{0.1}Ta_{0.9}O_{2.45}$	N/A	0.1 M $HClO_4$	*Nat. Energy* **7**, 55–64, (2022)
Ba[Co-POM]/carbon paste	24 h@0.35 mA cm^{-2} (from Fig. 3b)	1 M H_2SO_4 (pH <1)	*Nat. Chem.* **10**, 24–30, (2018)
$CoFeSbO_x$/FTO	160 h@10 mA cm^{-2} (from Supplementary Figure 13)	0.1 M H_2SO_4	*Nat. Catal.* **2**, 457–465 (2019)
$CoFeSbO_x$/Pt/Ti	6.8 h@500 mA cm^{-2} (from Supplementary Figure 23c)	1 M H_2SO_4	*Nat. Catal.* **2**, 457–465 (2019)
IrO_x/$SrIrO_3$	30 h@10 mA cm^{-2} (from Fig. 1a)	0.5 M H_2SO_4	*Science* **353**, 1011–1014 (2015)
Ir-W-B alloy rod (Φ 1 mm, solid rod)	120 h@100 mA cm^{-2} (from Fig. 2c)	0.5 M H_2SO_4	*Nat. Commun.* **12**, 3540 (2021)
Co_3O_4@C/GPO	43 h@10 mA cm^{-2} (from Fig. 4a)	1 M H_2SO_4 (pH 0.1)	*Nat. Commun.* **13**, 4341 (2022)
$Ru_5W_1O_x$/carbon paper (TGP-H-060, Toray)	550 h@10 mA cm^{-2} (from Fig. 2c)	0.5 M H_2SO_4 (pH 0.2)	*Nat. Commun.* **13**, 4871 (2022)
$Li_{0.52}RuO_2$/glassy carbon electrode	70 h@10 mA cm^{-2} (from Fig. 2d)	0.5 M	*Nat. Commun.* **13**, 3784

		H_2SO_4	(2022)
$Cr_{0.6}Ru_{0.4}O_2$/glassy carbon electrode	10 h@10 mA cm^{-2} (from Fig. 4e)	0.5 M H_2SO_4	*Nat. Commun.* **10**, 162 (2019)
$SrCo_{0.9}Ir_{0.1}O_{3-\delta}$/glassy carbon electrode	3 h@10 mA cm^{-2} (from Fig. 5b)	0.1 M $HClO_4$	*Nat. Commun.* **10**, 572 (2019)
3R-IrO_2/carbon paper	511 h@10 mA cm^{-2} (from Fig. 3c) ~45 h@100 mA cm^{-2} (from Fig. 3c)	0.1 M $HClO_4$	*Joule* **5**, 3221–3234 (2021)
1T-IrO_2/glassy carbon electrode	45 h@50 mA cm^{-2} (from Fig. 4g)	0.1 M $HClO_4$	*Nat. Commun.* **12**, 6007 (2021)
AD–HN–Ir/3D carbon paper	100 h@10 mA cm^{-2} (from Fig. 3f)	0.5 M H_2SO_4	*Nat. Commun.* **12**, 6118 (2021)
$Bi_{1.5}Er_{0.5}Ru_2O_7$/carbon paper	100 h@10 mA cm^{-2} (from Fig. 4d)	0.1 M $HClO_4$	*Nat. Commun.* **13**, *4106* (2022)
Ir-MoO_3 embedded by graphitic carbon layers/carbon paper	48 h@10 mA cm^{-2} (from Fig. 5e)	0.5 M H_2SO_4	*Nat. Commun.* **12**, *5676* (2021)
Co_3O_4/CeO_2/carbon paper	100 h@10 mA cm^{-2} (from Supplementary Figure 30)	0.05 M H_2SO_4	*Nat. Commun.* **12**, 3036 (2021)
$Co_{0.05}Fe_{0.95}O_y$/Ti foil	50 h@10 mA cm^{-2} (from Fig. 5b)<br>80 h@10 mA cm^{-2} (from Fig. 5b)	pH=0.3<br>pH=2	*Chem. Commun.* **55**, 5017–5020, (2019)
Co_3O_4/FTO	12 h@10 mA cm^{-2} (from Fig. 5b)	0.5 M H_2SO_4	*Chem. Mater.* **29**, 950–957, (2017)
$CoFePbO_x$/FTO	50 h@1 mA cm^{-2} (from Fig. 5b)	pH=2	*Chem. Sci.* **8**, 4779–4794, (2017)
$Ni_{0.5}Mn_{0.5}Sb_{1.7}O_y$/ATO	168 h@10 mA cm^{-2} (from Fig. 2a)	1 M H_2SO_4	*Energy Environ. Sci.* **10**, 2103–2108, (2017)
m-Fe_2O_3/Ti foil	6 h@10 mA cm^{-2} (from Fig. 9a)	0.5 M H_2SO_4 (pH=3)	*J. Catal.* **365**, 29–35, (2018)
c-Fe_2O_3/Ti foil	24 h@10 mA cm^{-2} (from Fig. 9a)	0.5 M H_2SO_4 (pH=3)	*J. Catal.* **365**, 29–35, (2018)

Supplementary Table 2. Some representative unconventional water electrolysis systems.

System	Substantial fundamental or methodological advance (Problems to be solved)	Benefits	Disadvantages	Ref.
Mixed Ni–Fe hydroxide (MNF) electrocatalysts	Electrocatalyst degradation/deactivation	An intermittent reduction methodology to revivify catalytic activity, Pulsed electrolysis takes only a short time (2 min) to reduce the catalyst intermittently	1. Limited current density (<200 mA cm^{-2}) 2. Suitable for alkaline environments only	*Nat. Catal.* **3**, 743–753 (2020)
In situ generated catalyst for acidic O_2 evolution	Electrocatalyst degradation/deactivation	Longer electrolysis under acidic conditions, adding Co^{2+}, Fe^{3+} and Pb^{2+} into the electrolyte to achieve a long-time acidic water electrolysis	1. Limited current densities 2. Toxic Pb^{2+}-based system may harm the environment and human health	*Nat. Catal.* **2**, 457–465 (2019)
$H_3PMo_{12}O_{40}$-based approach to water splitting	Gas mixing	Forming H_2 and O_2 in turn (only H_2 is produced in a certain period of time)	Waste of electricity power (e.g. Step 1 with no H_2 generation)	*Nat. Chem.* **5**, 403–409 (2013)
$H_4[SiW_{12}O_{40}]$–mediated water-to-H_2 production	Gas mixing	Separately forming H_2 and O_2 in space and time (no H_2-O_2 mixing issue)	Additional noble metal catalysts	*Science* **345**, 1326–1330 (2014)
Water electrolysis using recyclable $Ni(OH)_2$/NiOOH	Gas mixing	Forming H_2 and O_2 in turn	Waste of electricity power (e.g. Step 1 with no H_2 generation)	*Nat. Commun.* **7**, 11741 (2016)
Electrochemical–thermally activated chemical cycle	Gas mixing	Separately forming H_2 and O_2 in space and time (no H_2-O_2 mixing issue)	Additional heating treatments	*Nat. Energy* **4**, 786–795 (2019)
Membrane-free flow-type electrolysis	Issues with membrane-based cells	Membrane-free electrolysis with affordable Fe/Co bifunctional catalysts	More electrolyzer components & more cumbersome assembly than conventional electrolyzer	*Nat. Commun.* **12**, 4143 (2021)
Aldehyde oxidation–	Limited H_2	H_2 is produced on both sides of	O_2 evolution may occur at industrial-level current	*Nat. Catal.* **5**,

hydrogen evolution coupling	production yield	the electrolyzers	densities, requiring the maintenance of high-concentrations of organic reactants	66–73 (2022)
Membrane-less water electrolysis enabled by rose-petal-effect-mimetic interface	Issues with membrane-based cells	Membrane-less water electrolysis at 1 A cm^{-2}, with 61.5% electrolysis efficiency	The stability test took less than 3 h	*Joule* **7**, 1852–1866 (2023)
In situ cyclical electrode repair towards robust electrolysis	Electrocatalyst degradation/deactivation under HECs	1. Significantly improves electrode lifespan under HECs (for Ni foam, ~47-fold increases in electrolysis time) by the in situ electrode repair during H_2 synthesis<br>2. Rich adjustable parameters<br>2. a.c.-based electrosynthesis over d.c.-based electrosynthesis is the energy saved by avoiding the inevitable rectification power loss and reduction of the capital investment in the electrochemical part of the plant (*Curr. Opin. Electrochem.* **28**, 100712, (2021) & *J. Electrochem. Soc.* **138** 3678–3685 (1991))<br>3. This work may act as a critical reference/model for later studies that develop membraneless electrolysis systems, valuable a.c.-based electrolysis processes, AMC-based electrocatalytic systems, and AMC-based electrodeposition techniques for preparing advanced materials, etc.	1. Relatively high overpotential (possible corresponding solution in future work: developing more stable and highly conductive substrate with higher surface area)<br>2. H_2-O_2 mixing and water self-heating (possible corresponding solution in future work: lowering the frequency, if the frequency of alternating current is low enough, then it can be considered direct current), extending the time between polarity switch<br>3. Some FE is lost due to precipitation (possible corresponding solution in future work: (1) improving/adjusting diffusion and interaction of ions, such as from Co^{2+} to Co^{2+}-Na^{+} combo; (2) Reduce the ion concentration and find the optimal testing conditions)	*This work*
